# Synergistic Anti‐Cancer Therapy Through Ultrasound‐Induced Piezocatalytic Therapy and Tumor Treatment Fields

**DOI:** 10.1002/advs.202511604

**Published:** 2025-10-14

**Authors:** Jing Jin, Haoyue Xue, Keliang Chen, Yonglin Su, Xin Hu, Xiaolin Hu, Xing Huang, Laiming Jiang, Jiagang Wu, Xingchen Peng

**Affiliations:** ^1^ Department of Biotherapy Cancer Center West China Hospital Sichuan University Chengdu 610041 China; ^2^ Department of Obstetrics and Gynecology West China Second University Hospital of Sichuan University Chengdu Sichuan 610041 China; ^3^ Sichuan Provincial Key Laboratory of Nuclear Physics and Medical Research Sichuan University Chengdu 610041 China; ^4^ College of Materials Science and Engineering Sichuan University Chengdu 610064 China; ^5^ Department of Rehabilitation Cancer Center West China Hospital Sichuan University Chengdu 610041 China; ^6^ West China School of Nursing West China Hospital Sichuan University Chengdu 610041 China

**Keywords:** bioelectronics, cancer therapy, nanomedicines, piezocatalytic therapy (PCT), tumor treating fields (TTFs)

## Abstract

The integration of therapeutic modalities to enhance cancer treatment efficacy and improve patient outcomes offers remarkable benefits over monotherapies and has attracted considerable attention. Herein, a synergistic anti‐cancer therapy leveraging the benefits of physical tumor treating fields (TTFs) and dynamic piezocatalytic therapy (PCT) is presented, aiming to offer an optimized scheme while minimizing systemic side effects. First, a wearable ultrasound‐induced wireless energy harvester based on biocompatible lead‐free potassium sodium niobate‐based piezocomposites is developed to enable efficient acoustic‐to‐electric conversion. The flexible printed circuit board architecture with two pairs of half‐ring electrodes not only generates wide‐anging alternating electric fields but also facilitates easy fixation and wearability. Second, biocompatible piezoelectric nanoparticles (NPs) serve as the piezocatalysts to promote the generation of reactive oxygen species (ROS) and to directly interfere with biological processes. Finally, in vivo evaluations using mouse models demonstrate that the combination of ultrasound‐activated PCT and TTFs significantly inhibits tumor growth and modulates the tumor immune microenvironment. Notably, prominent synergistic antitumor effects are also observed in patient‐derived xenograft (PDX) models of head and neck squamous cell carcinoma (HNSCC), highlighting a versatile approach to advance cancer treatment.

## Graphical abstract

A novel, wearable anti‐cancer platform synergistically combines tumor treating fields (TTFs) and piezocatalytic therapy (PCT) via ultrasound‐activated, lead‐free piezocomposites. This strategy efficiently suppresses tumor growth, modulates the immune microenvironment, and demonstrates strong therapeutic synergy in both mouse model of subcutaneous tumor and PDX model of head and neck squamous cell carcinoma (HNSCC).

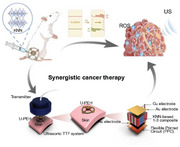

## 1 Introduction

Cancer is a multifaceted global health challenge.^[^ ^1^ ^]^ Cancer therapy has witnessed significant advancements with the emerging treatment modalities aimed at enhancing efficacy. Among these, tumor treatment fields (TTFs) therapy represents a promising approach, featuring non‐invasive, low toxicity, favorable efficacy, and highly sensitivity, which interferes with the mitotic mechanism of tumor cells by applying low‐intensity alternating electric fields, thereby inhibiting cell division and promoting apoptosis.^[^ ^2^, ^3^, ^4^ ^]^ TTFs has been approved by the Food and Drug Administration (FDA) for various solid tumors, including glioblastoma multiforme (GBM), where it has demonstrated efficacy in extending progression‐free survival (PFS)and overall survival (OS).^[^ ^2^, ^5^, ^6^ ^]^ Clinical trials for other localized solid tumors are ongoing, such as non‐small‐cell lung cancer and pancreatic adenocarcinoma.^[^ ^5^, ^6^, ^7^, ^8^ ^]^ However, its local therapeutic efficacy depends on the precise placement of electrodes, potentially limiting its applicability to diffusely infiltrative tumors. Moreover, challenges such as patient compliance with long‐term device wear and skin reactions underscore practical obstacles.^[^ ^9^ ^]^

Piezomaterials, characterized by their properties to convert electric to mechanical energy and vice versa, have found extensive applications in bioelectronic devices designed for implantable and wearable electrical stimulation, with promising outcomes in improving patient treatment and management.^[^ ^10^, ^11^, ^12^, ^13^, ^14^ ^]^ However, most piezoelectric devices are currently dominated by lead‐based piezoelectric materials, which pose environmental and health risks due to their toxicity. Their lead‐free alternatives address concerns regarding the biosafety, particularly for these bioelectronics intended for direct interaction with the human body, thereby advancing the current development of TTFs devices.^[^ ^10^, ^11^, ^13^ ^]^ Flexible, wearable TTFs devices based on such materials will be the focus of future research. Furthermore, piezocatalytic therapy (PCT) has emerged as a cutting‐edge strategy in the field of cancer treatment, leveraging the unique properties of biocompatible piezoelectric nanoparticles (NPs) to address the limitations of conventional therapies. Under ultrasound (US) stimulation, these NPs generate reactive oxygen species (ROS) through piezoelectric effects, offering a minimally invasive approach with more precise drug delivery capabilities.^[^ ^15^ ^]^ Compared to photodynamic therapy (PDT), US exhibits an overwhelming advantage in terms of deep tissue penetration and is able to target deep‐sited tumors. Unlike chemodynamic therapy (CDT), PCT triggers ROS generation on demand, thereby minimizing potential safety risks.^[^ ^16^, ^17^ ^]^ Collectively, PCT minimizes systemic adverse side effects and enhances antitumor efficacy, representing a significant advancement in next‐generation cancer therapeutics.

**Figure 1:**
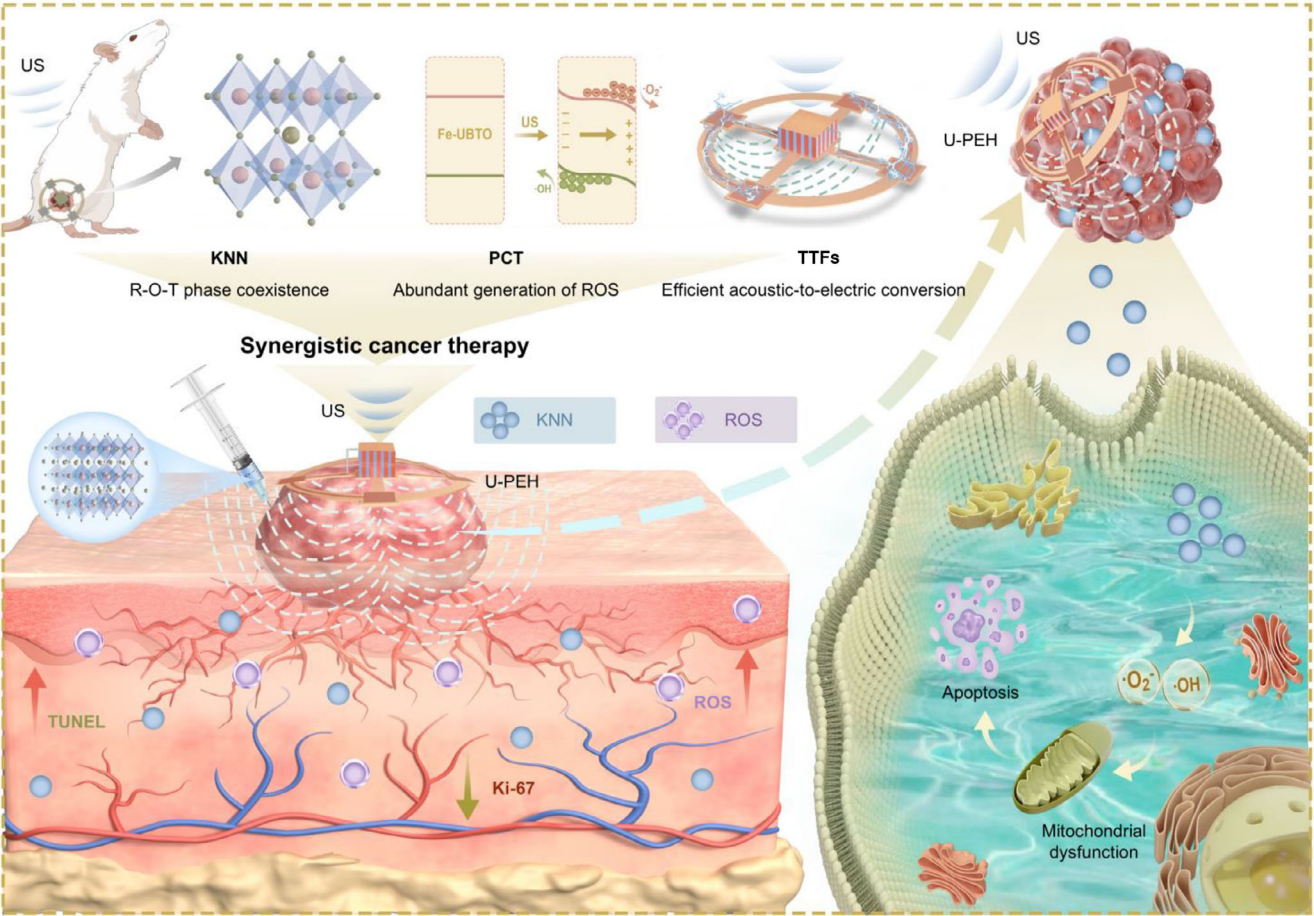
Schematic diagram of the design and principles of synergistic anti‐cancer therapy through US‐induced PCT and TTFs.

Aiming at optimize existing cancer therapies by leveraging the advantages of the aforementioned treatments, we have implemented a synergistic strategy combining US piezoelectric energy harvester (U‐PEH) for TTFs with injected biocompatible piezocatalysts for PCT (**Figure** **1**). The U‐PEH utilizes 1‐3 piezoelectric composites fabricated from high‐performance lead‐free potassium sodium niobate‐based lead‐free ceramics as the core coupling component, optimizing the conversion of acoustic energy into electrical energy.^[^ ^18^ ^]^ Featuring a flexible printed circuit board architecture with two pairs of half‐ring electrodes, the U‐PEH generates an alternating electric field that covers a wide area while ensuring easy fixation and enhanced wearability. The injected potassium sodium niobate‐based NPs with improved piezoelectric properties serve as piezocatalysts capable of catalyzing various redox reactions.^[^ ^19^, ^20^, ^21^ ^]^ As a result, upon US stimulation, the U‐PEH produces an alternating electric field for TTFs, and the activated NPs with built‐in electric field manipulate charge carrier migration and generate enhanced ROS, thereby directly interfering with critical biological processes. This dual synergistic strategy integrates dynamic and physical therapies to enhance therapeutic efficacy with spatial and temporal specificity, while minimizing systemic toxicity. In vitro and in vivo experiments demonstrated that the synergistic therapy effectively inhibited tumor cell proliferation and tumor growth, promoted tumor cell apoptosis, and regulated the tumor immune microenvironment. The minimal‐invasive nature of this synergistic technique further holds potential for outpatient management and improved patient compliance.

## 2 Results

### 2.1 Multilevel Structural Characterization and Piezocatalytic Activity of KNN NPs

**Figure 2:**
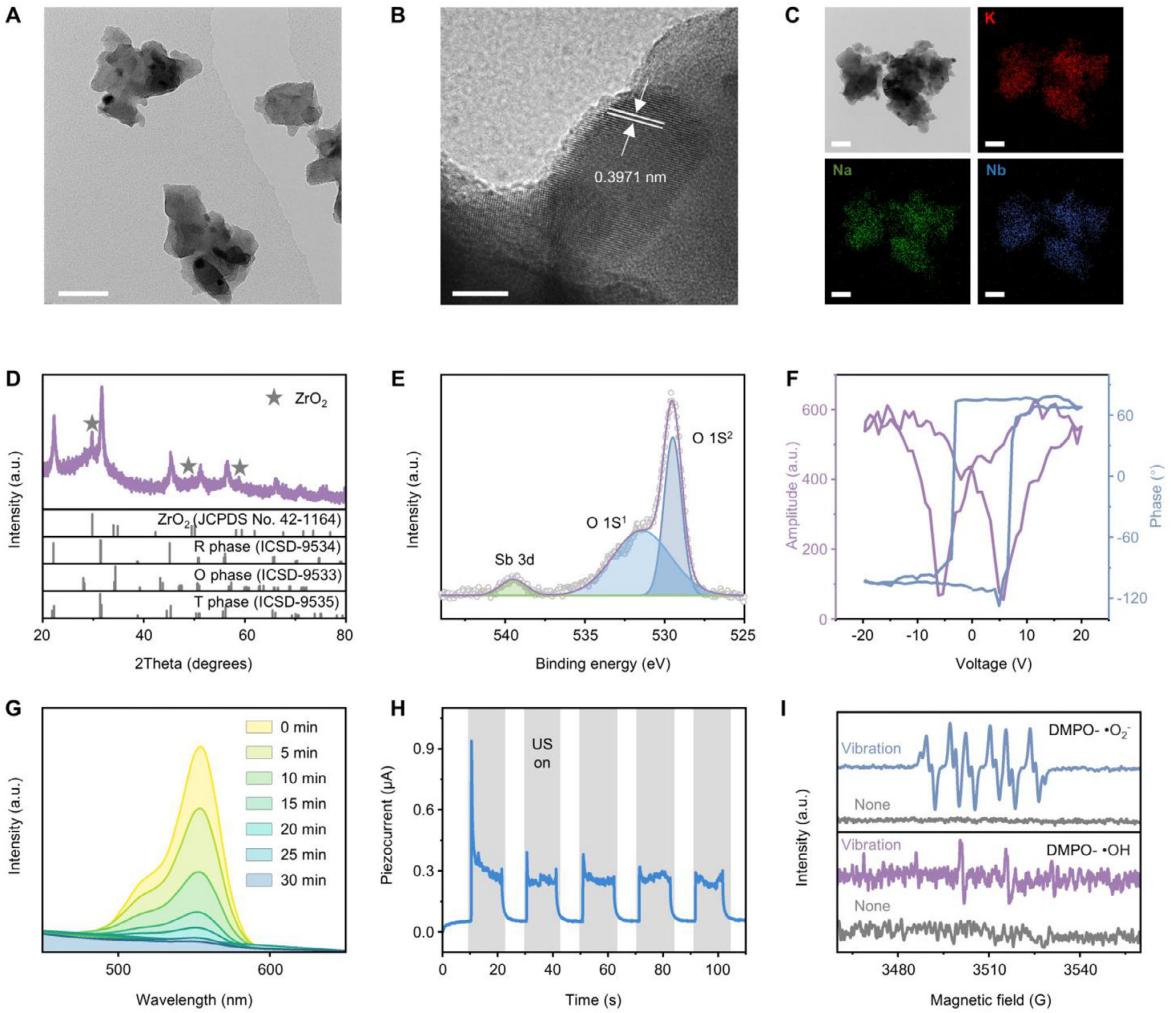
Structural and piezocatalytic characterizations of KNN NPs. A) TEM image of KNN NPs. Scale bar, 50 nm. B) HRTEM morphologies and lattice fringes of KNN NPs. Scale bar, 10 nm. C) TEM morphologies and corresponding element mapping images of KNN NPs. Scale bars, 100 nm. D) XRD patterns of KNN NPs. E) XPS spectra of Sb 3d and O 1s of KNN NPs. F) SS‐PFM measurements of KNN NPs. G) Degradation of Rho B by KNN NPs under US stimulation with increasing stimulation time from 0 to 30 min. H) Piezo‐current response of KNN NPs under US stimulation. I) EPR spectra of DMPO‐ •O_2_^−^ and DMPO‐ •OH over KNN NPs under US stimulation.

Piezoelectric activity is a pivotal parameter for the application of NPs for PCT. To enhance piezocatalytic performance, a lead‐free piezoelectric system of 0.905K_0.48_Na_0.52_NbO_3_‐0.055NaSbO_3_‐0.04Bi_0.5_Na_0.5_ZrO_3_‐0.2%Fe_2_O_3_ (abbreviated as KNN) with rhombohedral‐orthorhombic‐tetragonal (R‐O‐T) phase coexistence was selected for its extraordinary piezoelectric properties.^[^ ^22^ ^]^ The microstructure of KNN NPs was characterized using transmission electron microscopy (TEM), revealing that KNN NPs crystalized in irregular shapes with an average size of <100 nm (**Figure** **2**A). Clear and well‐ordered lattice fringes extended throughout individual grains with an interplanar distance of 0.3971 nm (Figure 2B). Elemental mapping demonstrated the relatively homogeneous distribution of all elements except for Zr element, despite the complex composition of KNN NPs (Figure 2C; Figure S1, Supporting Information). Dynamic light scattering (DLS) analysis revealed a particle size distribution of ≈250 nm and a zeta potential of −25.9 mV, with the larger hydrodynamic size compared to TEM observations attributed to the hydration layer and agglomeration in dispersion (Figure S2, Supporting Information). The X‐ray diffraction pattern of KNN NPs indicated a typical perovskite structure consistent with results from multiphase coexisting piezo‐ceramics (Figure 2D).^[^ ^23^ ^]^ Minor traces of ZrO_2_ (JCPDS data No. 42‐1164) were detected (Figure 2D), possibly due to the incomplete reaction during low‐temperature sintering,^[^ ^24^ ^]^ correlating with the enrichment of Zr observed in microstructures (Figure S1, Supporting Information). The chemical composition of the KNN NPs was further investigated using X‐ray photoelectron spectroscopy (XPS) analysis, with the full XPS spectra shown in Figure S3 (Supporting Information). All elements were clearly identified, and high‐resolution spectra for O 1s and Sb 3d along with their fitting curves, were presented in Figure 2E. The O 1s peak was decomposed into two distinct peaks, O 1s^1^ at 531.4 eV and O 1s^2^ at 529.5 eV, corresponding to oxygen vacancy and lattice oxygen, respectively.^[^ ^19^ ^]^ It has been reported that KNN‐based piezoelectric materials are typically inherited with abundant oxygen vacancies owing to the volatilization of alkaline elements during sintering.^[^ ^20^ ^]^ These oxygen vacancies further enhance molecular oxygen O_2_ dissociation, promoting ROS •O_2_^−^ production.^[^ ^21^ ^]^ The distinctive Sb 3d peak at 539.5 eV primarily indicated the incorporation of Sb^5+^ into the KNN lattice.^[^ ^23^, ^24^ ^]^ Furthermore, intrinsic piezoelectricity was investigated by the piezo‐response force microscopy (PFM) with switching spectroscopy (SS‐PFM) mode at the nanoscale. A typical butterfly‐shaped amplitude curve and a rectangular phase loop were observed (Figure 2F). The large displacement of ≈600 pm and the nearly 180° phase contrast demonstrated high piezoelectricity and excellent ferroelectricity of the as‐prepared KNN NPs, rendering their potential efficient piezocatalysts.

The piezocatalytic performance of KNN NPs was assessed through the degradation experiments of Rhodamine B (Rho B) under US stimulation. Experiments were conducted in darkness to prevent photocatalysis, and the water temperature in the ultrasonic bath was maintained within ± 1 °C to eliminate pyroelectric catalysis. A significant reduction in the absorption peak at 554 nm was observed, with near‐complete degradation of Rho B after 30 minutes of US stimulation (Figure 2G). Control experiments confirmed that US stimulation alone caused no significant degradation of Rho B, highlighting the pieocatalytic activity of KNN NPs (Figure S4, Supporting Information). Furthermore, distinct transient piezoelectric currents were observed upon switching the US stimulation on and off, indicating effective separation and transmission of free charges electrons and holes, which are conducive to piezocatalytic activity (Figure 2H).^[^ ^25^, ^26^ ^]^ To clarify the mechanism driving the piezocatalytic effect, the band structure of the KNN NPs was investigated using UV‐vis diffuse reflectance absorption spectra and valance band X‐ray photoelectron spectra (VB‐XPS) (Figure S5, Supporting Information). The UV‐vis spectra were obtained from the following Kubelka Munk Equation (1):

(1) $$F(R_{\infty })=K/S={\left(1-R_{\infty }\right)}^{2}/2R_{\infty }$$

where *K* is the adsorption coefficient and *S* represents the scattering coefficient, *R*_∞_ refers to the reflectance of a sample that is sufficiently thick to be considered optically infinite.^[^ ^27^ ^]^ The absorption edge (λ_edge_) of KNN NPs was observed at 316 nm, corresponding to a calculated band gap (*E*_g_) of 3.92 eV (Figure S5a, Supporting Information). Furthermore, the valance band maximum potential (*E*_VB_) was estimated to be ≈2.21 V versus the normal hydrogen electrode (NHE) (Figure S5b, Supporting Information). Therefore, the bottom of the conduction band (*E*_CB_) of the KNN NPs can be obtained by the formula as follows:^[^ ^28^ ^]^

(2) $$E_{\mathrm{CB}}=E_{\mathrm{VB}}-E_{g}$$

The calculated *E*_CB_ is −1.71 V (vs NHE). The band structure of the KNN NPs is illustrated in Figure S6 (Supporting Information). In conclusion, KNN NPs, whose *E*_CB_ (−1.71 V) is sufficiently negative to drive the O_2_/•O_2_^−^ oxygen reduction reaction at −0.33 V versus NHE and *E*_VB_ (+2.21 V) is sufficiently positive to promote the •OH/H_2_O oxidation reaction at +1.99 V versus NHE, can realize the thermodynamically favorable redox reaction.^[^ ^29^, ^30^ ^]^ Under US stimulation, the built‐in piezo‐potential field promotes continuous separation and transformation of charge carriers, leading to their accumulation toward surfaces with opposite charges and thereby generating ROS •OH and •O_2_^−^.^[^ ^31^ ^]^ Subsequently, these generated free radicals were detected by electron paramagnetic resonance (EPR) spin trap measurements, utilizing 5, 5‐dimethyl‐1‐pyrroline N‐oxide (DMPO) as the spin trapper. As shown in Figure 2I, distinct peaks corresponding to DMPO‐•O_2_^−^ and DMPO‐•OH were identified under US stimulation, thereby confirming the generation of •OH and •O_2_^−^ active species. Additionally, KNN NPs demonstrate robust structure stability under physiological conditions throughout the intended functional period (Figure S7, Supporting Information).

### 2.2 Validation of U‐PEH

**Figure 3:**
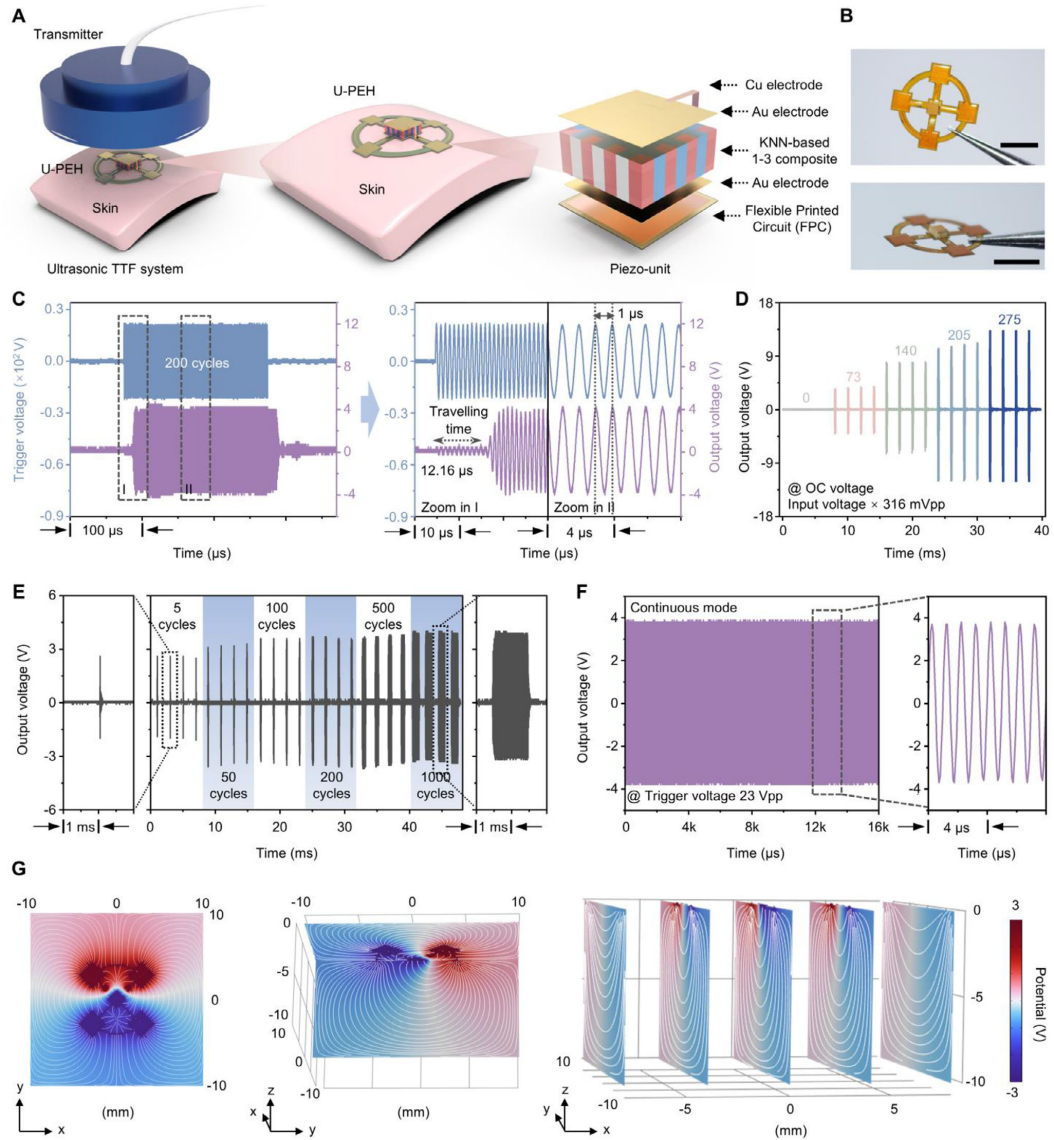
U‐PEH characterization. A) Schematic diagram of the ultrasonic TTFs system and its components. B) Optical photographs of the U‐PEH device. Size bars, 5 mm. C) Comparison between input trigger voltage and output voltage. D) Output voltages of the U‐PEH as the input voltage increases. E) Output voltages of the U‐PEH across various duty cycles (5‐1000). F) U‐PEH output voltages in continuous mode operation. G) FEA simulations of potential distribution near U‐PEH at an input voltage of 3 V.

The schematic diagram illustrating the TTFs treatment is depicted in **Figure** **3**A. Figure 3B shows optical images of the U‐PEH device, with a detailed description of its characterization and manufacturing processes provided in the Experimental Section. A 1‐MHz lead zirconate titanate (PZT) bulk transducer was chosen as the US transmitter for reliable and sufficient US transmission. Upon US waves reaching the U‐PEH, the core component, a KNN‐based 1‐3 piezo‐unit, directly converts acoustic energy into electrical outputs via the piezoelectric effect, ultimately generating alternative current signals with the same frequency as US waves.^[^ ^32^ ^]^ To evaluate the performance of U‐PEH, the trigger signal from the transmitter and the output signal generated by the U‐PEH under the corresponding US excitation were compared (Figure 3C). Both signals exhibited a sinusoidal waveform with a periodicity of 1 µs since the consistent 1 MHz frequency. It is worth noting that the output signal showed a slight time delay (≈12.16 µs) with a broader waveform, attributable to the travel in the aqueous medium and the reflection of US.^[^ ^33^, ^34^ ^]^ These results further demonstrated that the output signals are excited by the US waves. Figure 3D illustrated the open‐circuit (OC) output voltages of the U‐PEH measured under varying trigger voltages. The output voltage increased linearly with the trigger voltage, achieving a voltage efficient of ≈36% (Figure S8, Supporting Information). At a trigger voltage of 87 Vpp (peak‐to‐peak voltage), the output voltage reached 25.4 Vpp, sufficient for TTFs treatments as reported previously (typically less than 10 Vpp).^[^ ^4^, ^9^ ^]^ Additionally, the U‐PEH output can be flexibly tuned by modifying the duty cycles of the US to adapt to diverse treatment scenarios as required (Figure 3E). Continuous mode operation showed no significant attenuation in output voltage, indicating the stability of US transmission as well as the U‐PEH treating system (Figure 3F).^[^ ^33^, ^34^ ^]^ Finite element analysis (FEA) simulations were conducted to evaluate the potential distribution generated by the U‐PEH during TTFs therapy (Figure 3G). The results revealed that the U‐PEH produced a widespread potential distribution in three‐dimensional spatial, enabling efficient TTFs therapy. Additionally, the potential distribution of the U‐PEH can be precisely controlled by modulating the intensity of the US waves through adjustments to the trigger signal sent to the US transducer. This allows real‐time tuning of the electrical output, providing a flexible and efficient method for optimizing the local potential distribution and ensuring effective operation of TTFs therapy.

### 2.3 In Vitro Antitumor Performance of KNN NPs

**Figure 4:**
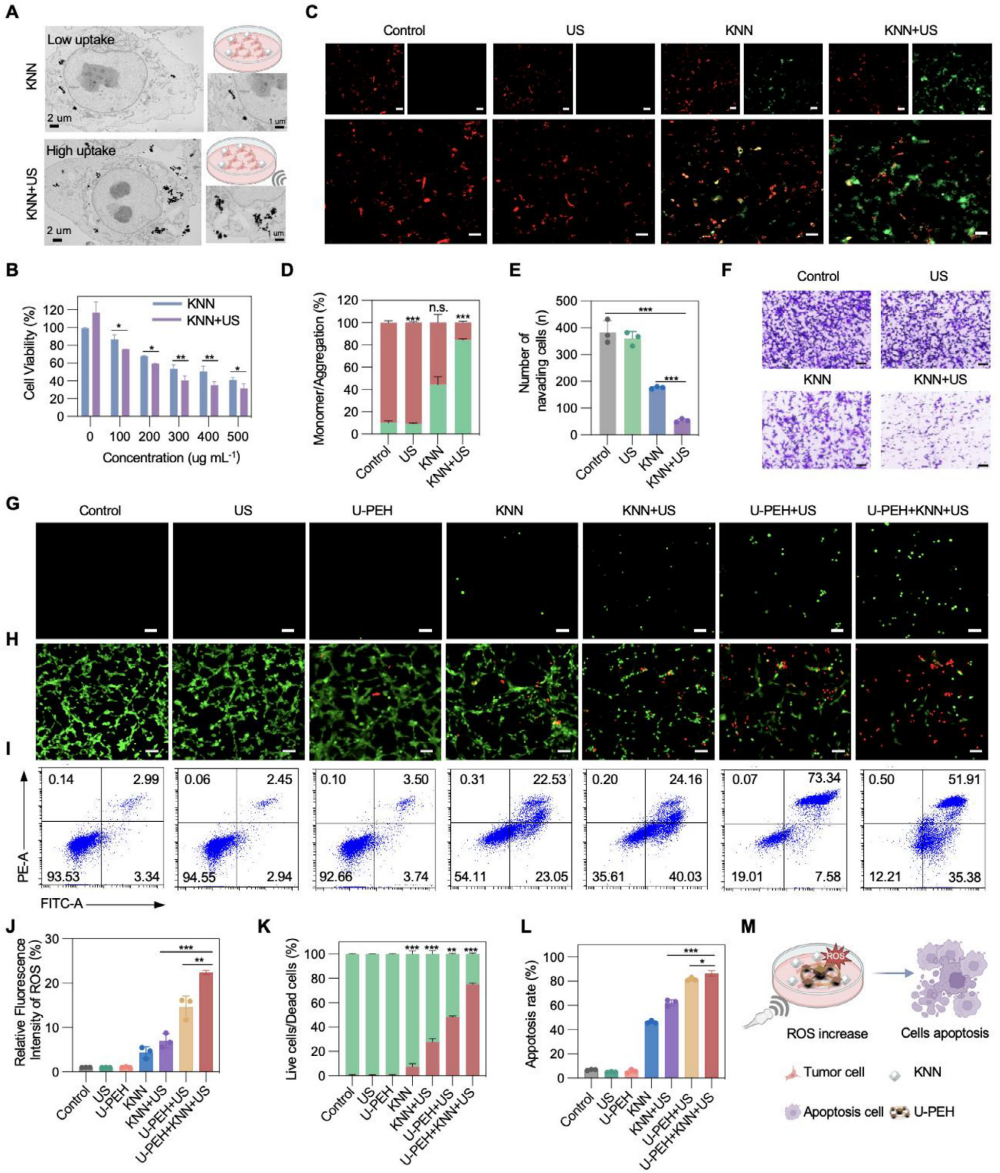
In vitro therapeutic efficacy of synergistic therapy. A) Bio‐TEM images of CT26 cancer cells following incubation with KNN NPs, with or without exposure to US stimulation. B) Cytotoxicity of KNN NPs on CT26 cells with US stimulation assessed by CCK8 assay. C) Fluorescence images of JC‐1 stained CT26 cells following various treatments. Scale bars, 100 µm. D) Green/red fluorescence ratios of JC‐1 stained CT26 cells following various treatments (n = 3). E) Quantification of Transwell assay of invasion ability of CT26 cells after different treatments (n = 3). F) Transwell assay of the invasion ability of CT26 cells after different treatments. Scale bars, 100 µm. G) Fluorescence images of DCFH‐DA in CT26 cells after different treatments. Scale bars, 50 µm. H) Live (green) and dead (red) assay of CT26 cells treated with KNN NPs with/without U‐PEH under US stimulation. Scale bars, 100 µm. I) Flow cytometry of apoptotic CT26 cells after treatment with control, US, U‐PEH, KNN, KNN+US, U‐PEH+US, and U‐PEH+KNN+US after 48 h. J) Quantitative analysis of DCFH‐DA in CT26 cells after different treatments (n = 3). K) Quantitative analysis of red/green fluorescence ratios in CT26 cells (n = 3). L) Corresponding quantification statistics of apoptosis in CT26 cells after 48 h different treatments. M) Schematic diagram of synergistic treatment. Created with BioRender.com. Data are presented as mean ± S.D. Statistical significance was calculated by two‐sided Student's t‐test. *p < 0.05, **p < 0.01, ***p < 0.001.

Encouraged by the efficient ROS generation of US‐activated KNN NPs, we investigated the biological efficacy and mechanism of KNN NPs against tumor cells. Initially, the intracellular uptake of KNN NPs was verified by bio‐TEM analysis. As shown in **Figure** **4**A, electron microscopy of CT26 cells after 4 h of treatment with KNN NPs confirmed the efficient internalization of the KNN NPs, and this effect was further enhanced by US stimulation. Next, we evaluated the cytotoxicity of KNN NPs on normal human immortalized epidermal cells (Hacat). Hacat cells exposed to high concentrations of KNN NPs (400 ug mL^−1^) exhibited ≈80% viability after 24 h, indicating low toxicity and favorable biosecurity of KNN NPs (Figure S9, Supporting Information). To assess the PCT efficacy of KNN NPs on mouse colon cancer cells CT26 and mouse squamous cell carcinoma cells SCC7, CCK8 assays were carried out and revealed concentration‐dependent cytotoxic effects. Specifically, the US‐activated KNN NPs exhibited IC50 values of 249 ug mL^−1^ in CT26 cells (Figure 4B), and 52.4 ug mL^−1^ in SCC7 cells (Figure S10, Supporting Information), respectively. Compared to previous studies demonstrating the effectiveness of KNN‐based PCT in inhibiting the proliferation of osteosarcoma cells,^[^ ^31^ ^]^ our findings indicated superior anti‐tumor effects of KNN NPs at lower concentrations due to their favorable piezoelectricity. JC‐1 staining analysis revealed excessive ROS production affecting mitochondrial membrane potential, which is indicative of mitochondrial malfunction.^[^ ^35^, ^36^ ^]^ Compared to other groups, KNN+US group exhibited stronger green fluorescence and a higher green/red fluorescence intensity ratio, suggesting synergistic mitochondrial membrane depolarization and damaged in CT26 (Figure 4C,D) and SCC7 cells (Figure S11A,B, Supporting Information). Transwell invasion experiments indicated a significant reduction in the invasive capacity of CT26 (Figure 4E,F) and SCC7 cells (Figure S12 A,B, Supporting Information) following the PCT of KNN NPs.

### 2.4 In Vitro Synergistic Anti‐Cancer Therapy

TTFs disrupts tumor cell division, inducing apoptosis in rapidly dividing cancer cells by exerting biophysical forces on charged and polarized molecules essential for cell division, preventing cellular proteins from moving to the correct positions, which has been shown to inhibit brain tumor growth.^[^ ^3^, ^37^ ^]^ Therefore, U‐PEH, generating alternating TTFs under US stimulation, was applied with KNN NPs in order to induce an enhanced synergistic anti‐cancer therapy. To validate the synergistic antitumor therapeutic effect of US‐activated U‐PEH and KNN NPs, intracellular ROS levels were assessed using 2′,7′‐dichlorodihydrofluorescein diacetate (DCFH‐DA), which is a non‐fluorescent compound and is rapidly oxidized by ROS to produce green fluorescent DCFH. Under US stimulation, both the U‐PEH treatment and the combined synergistic treatment of U‐PEH and KNN NPs exhibited significantly stronger green fluorescence, with negligible fluorescence observed in the control, US and alone U‐PEH groups in CT26 (Figure 4G) and SCC7 cells (Figure S13A, Supporting Information). Notably, the U‐PEH+KNN+US group showed the highest fluorescence intensity among all groups both in CT26 (Figure 4J) and SCC7 cells (Figure S13B, Supporting Information). Results from calcein acetoxymethyl (calcein‐AM) and propidium iodide (PI) staining showed that the US‐activated KNN NPs significantly promoted apoptosis in CT26 and SCC7 cells after 48 h of treatment. Dead cells were labeled with PI (red: dead cells), while the control, KNN, and US groups exhibited strong green fluorescence intensity with calcein‐AM (green: live cells), indicating relatively low cytotoxicity of KNN NPs and US stimulation on CT26 and SCC7 cells. Moreover, compared to the KNN+US group and U‐PEH alone group, both the U‐PEH+US and U‐PEH+KNN+US groups showed increased red fluorescence, with the U‐PEH+KNN+US group displaying the highest average intensity of red fluorescence in both CT26 (Figure 4H,K) and SCC7 cells (Figure S14A,B, Supporting Information). Furthermore, flow cytometry (FCM) assays elucidated the mechanism by which U‐PEH and KNN NPs synergistically induced apoptosis under US stimulation. The results indicated a substantial increase in apoptosis rates (up to ≈87%) in CT26 (Figure 4I,L) and SCC7 cells (Figure S15A,B, Supporting Information) with U‐PEH and KNN NPs after 48 h of co‐treatment with US stimulation, surpassing the rates in the U‐PEH+US and KNN+US groups. Conversely, minimal apoptosis was observed in the control group and the U‐PEH alone group, underscoring the synergistic pro‐apoptotic effect of the co‐treatment with U‐PEH and KNN NPs under US stimulation in antitumor therapy, demonstrating the pronounced cytotoxicity of the synergistic effect of TTFs and PCT (Figure 4M).

### 2.5 In Vivo Synergistic Anti‐Cancer Therapy

**Figure 5:**
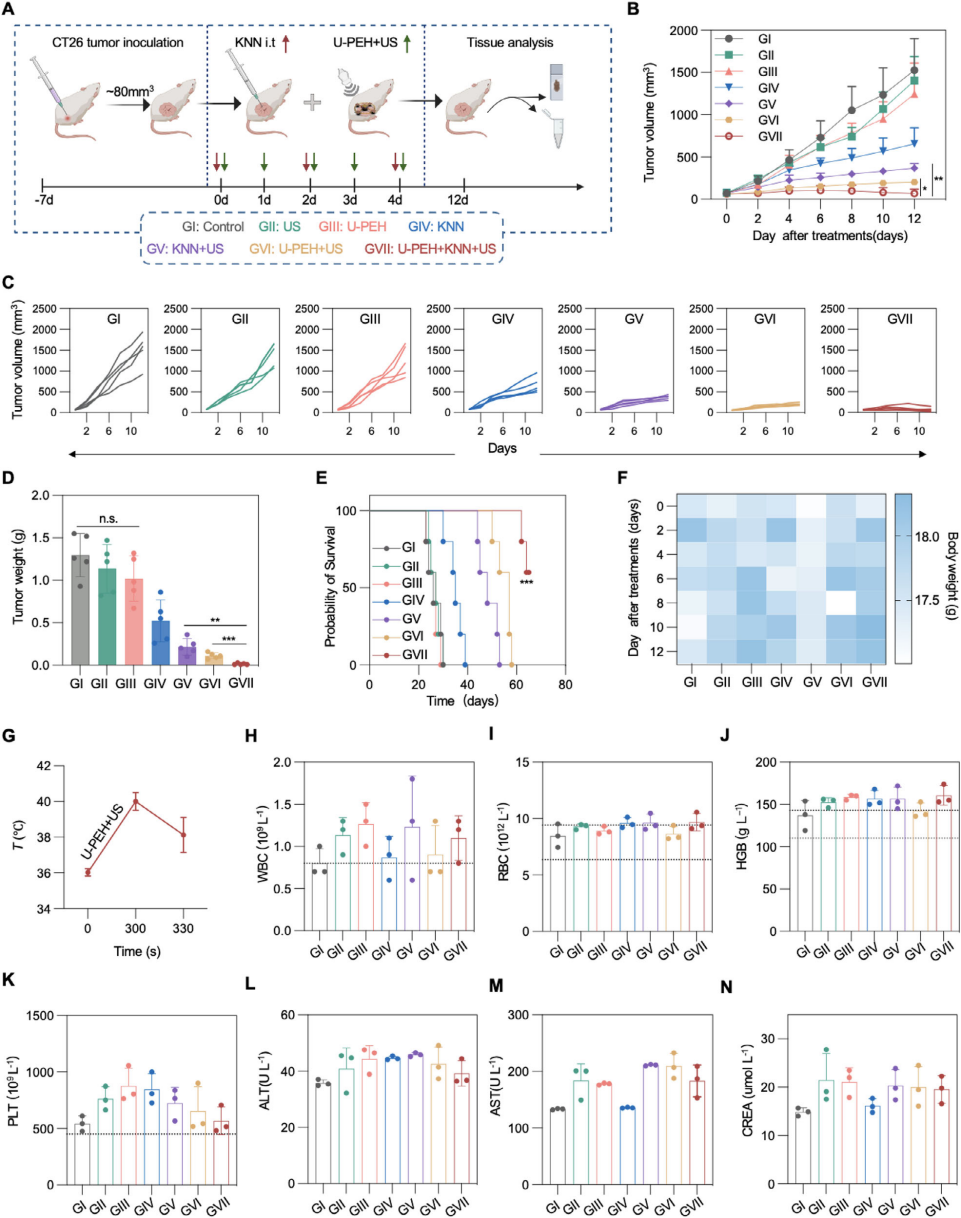
In vivo therapeutic efficacy of synergistic therapy of CT26 tumors. A) Timeline of interventions and assessments in CT26 tumor‐bearing mice. Created with BioRender.com. Average B) and individual C) tumor growth kinetics per group (n = 5). D) Resected tumor weights in Control, US, U‐PEH, KNN, KNN+US, U‐PEH+US, and U‐PEH+KNN+US groups from CT26 tumor‐bearing mice. E) Survival rates of CT26 tumor‐bearing mice (n = 5). F) Body weights of mice in diverse groups (n = 5). G) Tumor temperature profile of mice after U‐PEH+US treatment (n = 3). H–K) Routine blood analysis (range within the black dotted line indicates normal), including WBC H), RBC I), HGB J), and PLT K). L–N) Serum biochemical analysis of CT26 tumor‐bearing mice 12 days after different treatment, including ALT L), AST M), and CREA N). Data are presented as mean ± SD. Statistical significance was calculated by two‐sided Student's t‐test. Statistical analysis was performed via log‐rank test. *p < 0.05, **p < 0.01, ***p < 0.001.

We proceeded to evaluate the anti‐tumor effects of US‐activated co‐treatment of U‐PEH and KNN NPs in vivo using a CT26 xenograft tumor model. When the tumor size reached ≈60–80 mm^3^, mice harboring CT26 tumors were randomly divided into seven groups: (I) Control, (II) US, (III) U‐PEH, (IV) KNN, (V) KNN+US, (VI) U‐PEH+US, and (VII) U‐PEH+KNN+US. In the U‐PEH+KNN+US group, tumors were treated with U‐PEH under US stimulation over the initial five days of treatment (**Figure** **5**A). Analysis of tumor growth curves during the treatment period revealed rapid tumor growth in the control, US and alone U‐PEH groups. The KNN+US group showed potent inhibition of tumor growth due to piezocatalytic effect (Figure 5B,C). In contrast, the tumor inhibitory effects were significantly enhanced in the U‐PEH+US and U‐PEH+KNN+US groups, with the highest tumor suppression observed in the U‐PEH+KNN+US group. This rate was obviously higher than that in the KNN+US group, highlighting the enhanced synergistic antitumor effect of US‐activated U‐PEH and KNN NPs (Figure 5D; Figure S16, Supporting Information). Meanwhile, TEM analysis of tumor tissues after in vivo treatment with KNN NPs showed that the KNN NPs were evenly distributed within the tumor tissue (Figure S17A, Supporting Information). The niobium (Nb) content in tumors after a single KNN treatment was measured over time by inductively coupled plasma atomic emission spectroscopy (ICP‐OES), and the results indicated that KNN NPs maintained a high concentration in the tumor during the treatment period, followed by a gradual decrease due to metabolism (Figure S17B, Supporting Information). Survival analysis further demonstrated that U‐PEH+KNN+US synergistic treatment effectively prolonged mouse survival and sustained tumor suppression compared to other groups (Figure 5E).

The in vivo biosafety of the combination therapy was thoroughly evaluated during the treatment period. Notably, there were no significant fluctuations in the weights of the mice during the treatment (Figure 5F), and hematoxylin and eosin (H&E) staining of major organs (heart, liver, spleen, lung and kidney) exhibited no obvious toxic side effects, indicating the high biological safety of the treatment approaches (Figure S18, Supporting Information). Note that during operation of the TTFs system, the temperature variations were minimal, thus excluding thermal effect and no skin damage occurred (Figure 5G; Figure S19, Supporting Information). We further performed routine blood tests and serum biochemistry analyses to assess the biosafety of the combination therapy. As shown in Figure 5H–N, there were no significant differences in routine blood tests[white blood cells (WBC) (Figure 5H), red blood cells (RBC) (Figure 5I), hemoglobin (HGB) (Figure 5J), and platelets (PLT) (Figure 5K)] and blood biochemical parameters[alanine aminotransferase (ALT) (Figure 5L), aspartate aminotransferase (AST) (Figure 5M), and creatinine (CREA) (Figure 5N)] in all treatment groups, indicating the biosafety of the U‐PEH+KNN+US synergistic treatment.

**Figure 6:**
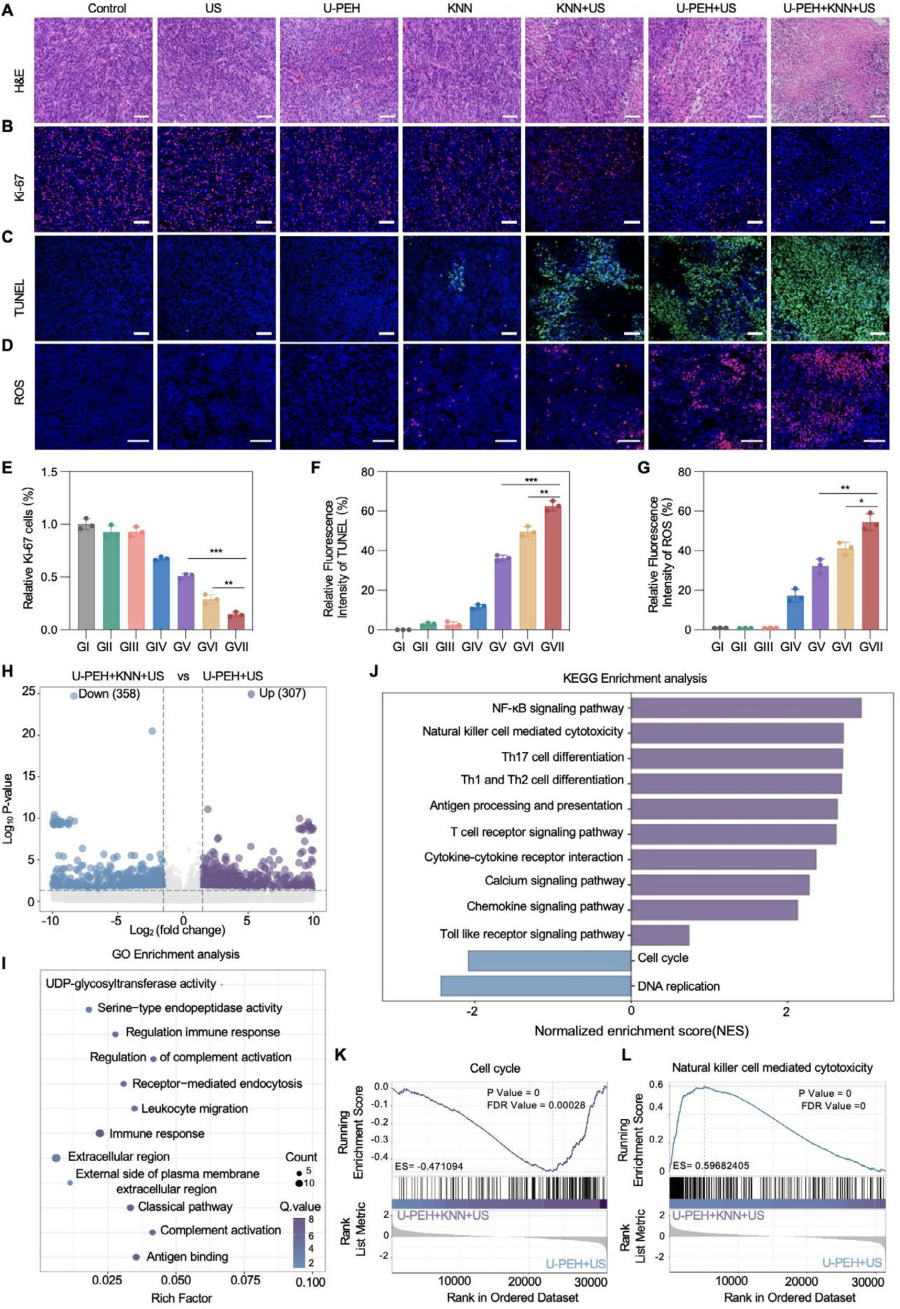
In vivo cancer therapy mechanism of synergistic therapy. A) H&E‐stained slices from mice with CT26 tumors following different treatments indicated. Scale bar, 50 µm. B) Representative images of Ki‐67 immunofluorescence staining in tumor slices. Scale bar, 50 µm. C) Representative immune‐fluorescence images of tumor slices stained with the TUNEL assay kit. Scale bar, 50 µm. D) Representative images of DHE staining as an indicator of ROS in each group. Scale bars, 100 µm. (E‐G) Calculation of the relative Ki‐67 cells E), mean fluorescence intensity of TUNEL F), and relative ROS fluorescence intensity G) following various treatments (n = 3). H) Volcano plots of all DEGs between U‐PEH+KNN+US and U‐PEH+US treatments. I) Analysis of the Gene ontology (GO) term enrichment among DEGs following U‐PEH+KNN+US treatment compared to U‐PEH+US treatments. J) Histogram of KEGG pathway enrichment of DEGs after U‐PEH+KNN+US treatment versus U‐PEH+US treatment. K,L) GSEA enrichment analysis of DEGs following U‐PEH+KNN+US treatment versus U‐PEH+US treatment. Data are presented as mean ± SD. The two‐sided Student's t‐test was utilized to determine statistical significance. *p < 0.05, **p < 0.01, ***p < 0.001.

Tumors from different treatment groups were subjected to immunohistochemistry and fluorescence staining to evaluate proliferative ability and verify the synergistic antitumor effect. Pathological H&E staining showed extensive tumor necrosis in residual tissue from the U‐PEH+KNN+US group (**Figure** **6**A), and Ki‐67 staining demonstrated significantly inhibited tumor cell proliferation (Figure 6B,E). The KNN+US group exhibited stronger green fluorescence in the TUNEL assay compared to the KNN group, suggesting the induced tumor cell apoptosis through KNN NPs‐mediated PCT (Figure 6C,F). However, the U‐PEH+KNN+US group showed stronger red fluorescence and effectively increased intratumoral ROS levels, indicating the synergistically antitumor effect induced by U‐PEH and KNN NPs under US stimulation (Figure 6D,G). Furthermore, to elucidate the molecular mechanism underlying the US‐activated co‐treatment of U‐PEH and KNN NPs on local tumor growth, we performed transcriptomic analysis on tumors from the U‐PEH+KNN+US and U‐PEH+US groups after 12 days of treatment. Specifically, we generated volcanic maps comparing U‐PEH+KNN+US and U‐PEH+US treatments, identifying a total of 665 different genes, including 307 up‐regulated genes and 358 down‐regulated genes (Figure 6H). Gene Ontology term enrichment study comparing the U‐PEH+KNN+US and U‐PEH+US groups revealed that the differentially expressed genes (DEGs) were primarily enriched in immune response, complement activation, classical pathway (Figure 6I). In addition, Kyoto Encyclopedia of Genes and Genomes (KEGG) pathway analysis highlighted over‐representation of genes involved in the NF‐κB signaling pathway, cell cycle, and DNA replication (Figure 6J). Gene set enrichment analysis (GSEA) was employed to further investigate the functional significance of these DEGs. The results revealed the inhibition of cell cycle progression (Figure 6K) and the activation of pathways related to natural killer (NK) cell‐mediated cytotoxicity (Figure 6L), antigen processing and presentation (Figure S20A, Supporting Information), and cytokine‐cytokine receptor interactions (Figure S20B, Supporting Information) in the U‐PEH+KNN+US co‐treatment induced tumor regression.

### 2.6 Synergistic Treatment in Patient‐Derived Xenograft (PDX) Models

**Figure 7:**
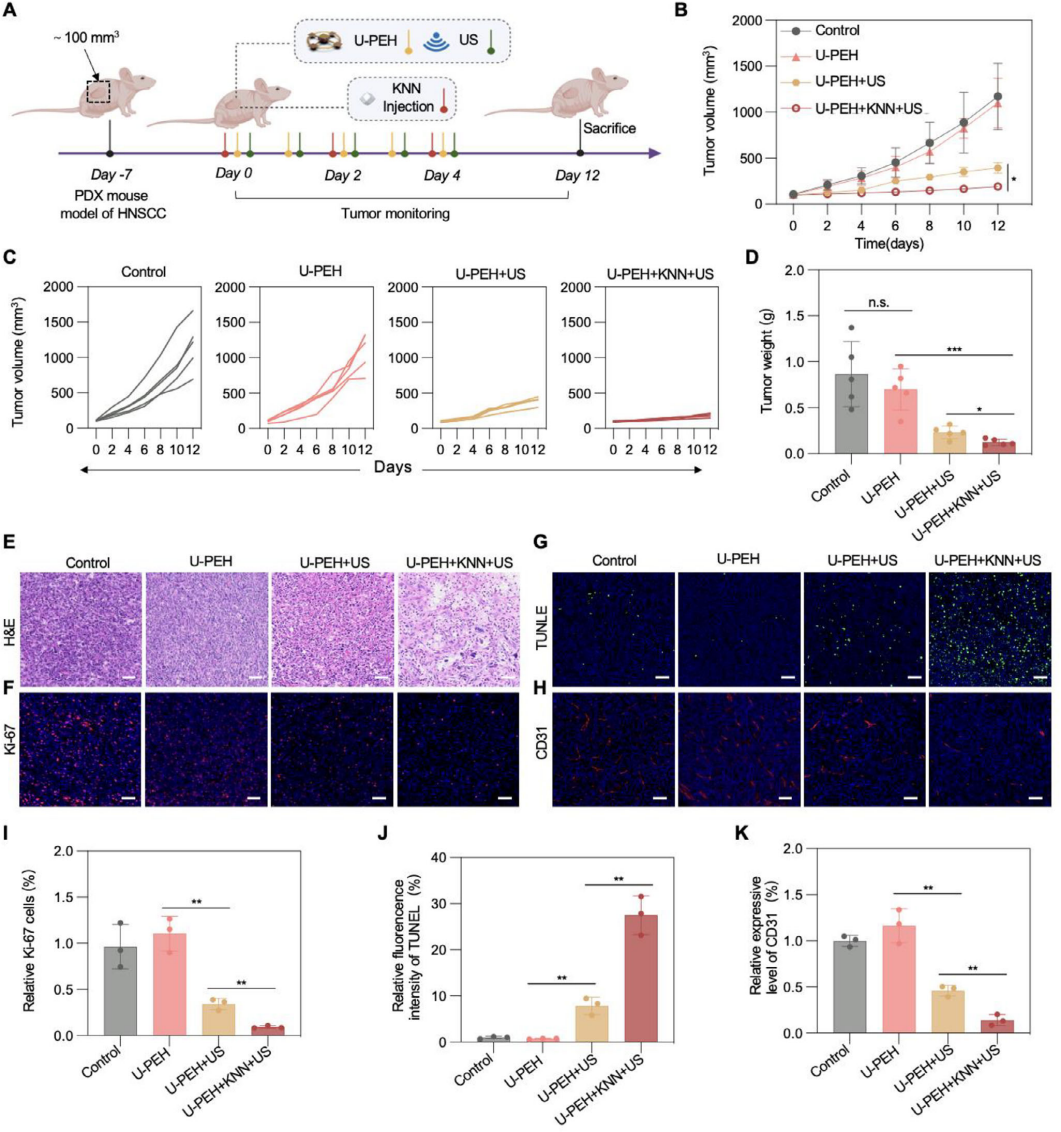
Therapeutic efficiency of U‐PEH+KNN+US in a PDX model of Head and neck squamous cell carcinoma(HNSCC). A) A schematic representation of the PDX mouse model's therapy regimen. Created with BioRender.com. B,C) Average B) and individual C) mouse tumor volumes in HNSCC PDX‐bearing mice with different treatments. D) Tumor weights in Control, U‐PEH, U‐PEH+US, and U‐PEH+KNN+US groups excised tumors from PDX model mouse. E) H&E staining of mouse tumor slices after different treatments. Scale bar, 50 µm. F–H) Ki‐67 F), TUNEL G), and CD31 H) staining of mouse tumor slices after different treatments. Scale bar, 50 µm. I–K) Quantification of the relative Ki‐67 positive cells I), TUNEL J), and CD31 K) mean fluorescence intensity after different treatments. Data are presented as mean ± SD. The two‐sided Student's *t*‐test was utilized to determine statistical significance. *p < 0.05, **p < 0.01, ***p < 0.001.

Encouraged by the synergistic therapeutic effect of KNN NPs and U‐PEH under US induction on subcutaneous tumor‐bearing mice, we evaluated the therapeutic potential of U‐PEH+KNN+US using a clinically relevant patient‐derived cancer xenograft (PDX) mouse model of head and neck squamous cell carcinoma (HNSCC). Mice in the U‐PEH+KNN+US group (n = 5 per group) carrying PDX tumors were intratumorally injected with KNN NPs at a dose of 10 mg kg^−1^ on days 0, 2, and 4. At the same time, TTFs is given on days 0 to 4 in both U‐PEH+US group and U‐PEH+KNN+US group. Monitor changes in tumor size and mouse body weight up to day 12 (**Figure** **7**A). During treatment monitoring, there was no notable change in the body weight of the mice throughout the course of the medication (Figure S20, Supporting Information), and no damage to major organs was observed, suggesting minimal adverse effects (Figure S21, Supporting Information). After 12 days, U‐PEH+US alone effectively inhibited tumor growth compared to mice in the control and U‐PEH alone groups. However, mice treated with U‐PEH+KNN+US showed more significant inhibition of tumor growth, and the stripped tumor weight also confirmed that the combined PCT and TTFs under US induction exhibited considerable tumor inhibition (Figure 7B–D). H&E staining assay in the U‐PEH+KNN+US treatment group showed extensive tumor necrosis and apoptosis (Figure 7E). Immunofluorescence data showed that the U‐PEH+KNN+US group significantly reduced Ki‐67 (Figure 7F,I) and increased TUNEL (Figure 7G,J) compared with the other groups, indicating that the combination therapy could effectively inhibit tumor proliferation and increase apoptosis. Simultaneously, the U‐PEH+KNN+US group had the greatest inhibition of tumor vascular growth, as demonstrated by anti‐CD31 antibody staining in the tumor vasculature (Figure 7H,K). Collectively, U‐PEH+KNN+US can reduce systemic toxicity and increase therapy efficacy for PDX tumors.

## 3 Discussion

In summary, we present a synergistic approach to anti‐cancer therapy combining PCT and TTFs under US stimulation. Specifically, we utilized high‐performance biocompatible lead‐free KNN NPs, which exhibited enhanced ROS generation, as desirable piezocatalysts. Meanwhile, the flexible U‐PEH, characterized by stable and adjustable outputs, facilitating efficient acoustic‐to‐electric conversion and easy manipulation. Under US stimulation, KNN NPs generated abundant ROS, while the alternating electric field generated by U‐PEH was effective for TTFs treatment. In vivo mouse subcutaneous tumor model and PDX tumor model studies validated that this enhanced synergistic therapy induced by US‐activated co‐treatment of KNN NPs and U‐PEH effectively induced tumor cell apoptosis and inhibited tumor cell growth. Furthermore, RNA sequencing analysis further underscored the increased efficacy of this dual therapy in modulating the tumor immune microenvironment and cytokine‐cytokine receptor interactions. This work represents a promising strategy for advancing cancer therapy and improving patient outcomes and quality of life.

Despite promising preclinical results, further efforts are essential to optimize treatment protocols for different tumors, including refining delivery methods for piezocatalysts and updating device architecture. It is crucial to continue refining formulations and properties of current NPs and even develop new catalysts to maximize therapeutic efficacy. Optimization of TTFs device structures for longer wear through stretchable and adhesive hydrogel design. Deep investigations into the mechanism of this synergistic therapy are also necessary to enhance specificity and reduce off‐target effects. Exploring synergies with other therapeutic modalities, such as chemotherapy or immunotherapy, could further enhance treatment outcomes, promote sequential or concurrent therapies targeting different aspects of tumor biology, and overcome resistance mechanisms. Incorporating advancements in biochemical detection technologies, imaging techniques, and bioengineered materials could lead to the development of next‐generation therapeutic platforms capable of real‐time monitoring of treatment response and adaptive therapeutic strategies tailored to individual patients. Clinical research also needs to be advanced for future clinical translation. Collectively, this synergistic therapy is expected to bring substantial benefits to cancer patients.

## 4 Experimental Section

### Preparation for KNN NPs

The 0.905K_0.48_Na_0.52_NbO_3_‐0.055NaSbO_3_‐0.04Bi_0.5_Na_0.5_ZrO_3_‐0.2%Fe_2_O_3_ (abbreviated as KNN) samples were fabricated using the solid‐state method. First, K_2_CO_3_ (99%), Na_2_CO_3_ (99.8%), Nb_2_O_5_ (99.95%), Sb_2_O_3_ (99.99%), ZrO_2_ (99%), Bi_2_O_3_ (99.5%), Fe_2_O_3_ (99%) (Sinopharm Group Co., Ltd, China) were weighted based on stoichiometric ratio. These materials were then ball‐milled with alcohol and ZrO_2_ balls in nylon jars for 24 h. After drying, the powders were calcined at 650 °C for 2 h. Finally, to further reduce the particles size, the calcined powders were subjected to sand‐grounded (2000 RPM, VB‐0.3Q, China) for 8 h.

### Preparation for KNN Ceramics

The ball‐milled and dried powders were calcined at 850 °C for 6 h. Following calcination, the powders were mixed with 8 wt.% polyvinyl (PVA) and pressed into pellets with dimensions of 10 mm in diameter of 10 and 1 mm in thickness, under a pressure of 10 MPa. The green pellets were then heated slowly to 850 °C to remove PVA. Finally, the pellets were sintered at 1080 °C for 3 h to obtain KNN ceramics.

### U‐PEH Preparation

A dicing‐filling technology (DS9260) was first employed to prepare the KNN/epoxy (ELINOPTO E106‐7 A/B) 1‐3 composites. Then the fabricated composites were mechanically polished and sputtered with Au electrodes by a sputtering system (TPR450). Next, the composites were cut into cubes with the volume of 2.5 mm × 2.5 mm × 1 mm and poled by applying a direct current electric field of 2 kV mm^−1^ for 15 min. The poled composite was connected to the center of the flexible printed circuit (FPC) using conductive silver paste. Then top of the cubic composite with Au electrode were connected to the other half of the FPC by Cu wire.

### Structural Characterization

The surface morphology and lattice fringes were characterized using TEM (Thermo Scientific Talos F200X). The crystal structure of KNN NPs was collected by XRD with Cu Kα radiation (Bruker D8 Advanced XRD, Bruker AXS Inc., Madison, WI, USA). XPS (AXIS Ultra DLD) was employed to investigated the surface valence states. UV‐vis diffuse reflectance spectra were obtained using a UV‐vis spectrophotometer (UV‐3600, Hitachi) was used to obtain the. Switching spectroscopy PFM experiments were conducted with a piezoelectric force microscopy (PFM, MFP‐3D) at an applied voltage of 20 V. Transient piezo‐current was recorded by an electrochemical workstation (CHI660E, China) in a three‐electrode system using 0.1 m Na_2_SO_4_ as the electrolyte. The working, reference, and counter electrodes consisted of FTO glass coated with KNN NPs, an Ag/AgCl electrode, and a platinum electrode, respectively. During the measurement, mechanical vibrations at a frequency of 40 kHz were generated using an ultrasonic machine with the validation of active radicals was performed by the electron paramagnetic resonance (EPR) experiments.

### Piezocatalytic Activity Characterization

The piezocatalytic activities were evaluated through the degradation experiments of Rho B under US stimulation (40 kHz, 120 W). In this process, 0.1 g of KNN NPs was added into 50 mL of Rho B solution (5 mg L^−1^) and stirred in the dark for 30 min to reach adsorption‐desorption equilibrium. Following this, the mixture was transferred to the ultrasonic machine for degradation experiments. At the time interval of 5 min, 3 mL of the mixture was sampled and centrifuged to obtain the supernatant. The concentration of Rho B in the obtained supernatant was subsequently examined by the UV‐vis spectrophotometer (UV‐1800 PC, MAPADA, China).

### Ultrasound‐Induced Electrical Output Measurement

The ultrasound‐induced output of the U‐PEH was measured with a multifunctional ultrasonic test platform. To start with, an input signal was produced by a function generator (AFG1062, Tektronix). After being amplified by a power amplifier (SSPA1M100M‐100N), the signal was further transferred to activate the 1 MHz US transmitter. The U‐PEH was placed under the transmitter for signal receiving. Output voltages generated by the U‐PEH were directly recorded with a digital oscilloscope (TBS 2000 Series). To ensure optimal US transmission, the experiments was taken out in deionized water.

### Finite Element Analysis

FEA simulations of the potential distribution of U‐PEH were conducted using COMSOL software (COMSOL Multiphysics 6.1). Relevant physical parameters for the simulations were derived from experimental measurements or manufacturer specifications. The model was designed to closely replicate the physical structure of the device, with careful attention given to accurately representing the relevant physical domains.

### Cells and Animals

CT26 murine colon cancer cell line (RRID: CVCL_7255) and SCC‐7 murine head and neck squamous cancer cell line (RRID: CVCL_V412) were kindly provided by the State Key Laboratory of Biotherapy, Sichuan University. Hacat cells (RRID: CVCL_0038) are derived from the Laboratory of Radiation Medicine, School of Basic Medical Sciences, Sichuan University. 6‐8 weeks Balb/c female mice and 3‐4 weeks Balb/c female nude mice were purchased from Jiangsu Jicui Yaokang Co., Ltd. Mice are housed in a standard barrier animal facility at Sichuan University. All animal experimental studies were approved by the Institutional Animal Care and Use Committee of Sichuan University (Approval No: 20 240 514 003).

### Cellular Uptake Experiment

CT26 cells were seeded in 6‐well plates and allowed to attach overnight. KNN NPs were added and incubated with the cells for 4 h. Afterwards, the cells were exposed to US stimulation (1.0 MHz, 1.5 W cm^−2^), then further incubated for a designated period. Finally, the cells were collected, fixed, and prepared for bio‐TEM (JEM‐1400FLASH, JEOL) to assess the cellular uptake of KNN NPs.

### Cytotoxicity Measurement

CT26 or SCC7 cells were cultivated in RPMI 1640 medium, and Hacat cells were cultured in Dulbecco's Modified Eagle's Medium (DMEM) containing 1% penicillin‐streptomycin and 10% fetal bovine serum at 37 °C in a 5% CO_2_ incubator. To evaluate the cytotoxicity of KNN, the CCK8 assay was employed. Cells (5 × 10^3^ cells per well) were cultured in 96‐well plates. For CT26 cells, various concentrations of KNN NPs (0, 100, 200, 300, 400, and 500 µg mL^−1^) were co‐incubated for 4 h. SCC7 cells were treated with KNN NPs at concentrations of 0, 25, 50, 100, 200, and 400 µg mL^−1^ under the same conditions. Hacat cells were exposed to KNN NPs at concentrations of 0, 50100, 200, 300, and 400 µg mL^−1^. Following co‐incubation, US (1.0 MHz, 1.5 W cm^−2^) was applied for 30 s, and cells were then further incubated for 24 h. CCK8 assays were conducted according to standard protocols, and absorbance was measured using a microplate reader.

### Apoptosis Assay

CT26 or SCC7 cells were seeded in six‐well plates at a density of 1 × 10^5^ cells per well and incubated for 24 h, respectively. Subsequently, cells were treated with different concentrations of KNN NPs (200 ug mL^−1^ for CT26 200 ug mL^−1^ and 100 ug mL^−1^ for SCC7) for 4 h. Following this treatment, the U‐PEH was placed in the well plate, and US (1.0 MHz, 1.5 W cm^−2^) was applied for 30 s. After treatment, cells were stained using the Fluorescein Isothiocyanate (FITC) Annexin V Apoptosis Assay Kit and quantified by FCM 48 h later.

### Transwell Assay

For the treatment of CT26 or SCC7 cells, the procedure mirrored that of the lived/dead cell staining assay. After 4 h of exposure to KNN NPs, cells underwent US treatment. Each group of cells was then harvested and seeded into the upper chamber of a Transwell system at a density of 8 × 10^4^ cells per well. The upper chamber contained 1640 medium without FBS, while the lower chamber contained 1640 medium with 10% FBS. After an additional 24 h of incubation in the incubator, CT26 or SCC7 cells at the bottom of the polycarbonate membrane were fixed with 4% paraformaldehyde and stained with 0.1% crystal violet. Cell invasion was observed with an optical microscope (Olympus digital camera, Olympus, Japan).

### Fluorescence Imaging

The following fluorescence images were photographed using an optical microscope (Olympus digital camera, Olympus, Japan).

For the JC‐1 staining assay, CT26 or SCC7 cells were seeded in 6‐well plates at a density of 1 × 10^5^ cells per well and incubated for 24 h. After 4 h of incubation with different concentrations of KNN NPs, US combined with U‐PEH were further treated for 30s. The culture was continued for an additional 24 h. Cells were stained with JC‐1 (5 µg mL^−1^) for 15 min and washed three times with PBS before imaging.

For the detection of intracellular ROS, CT26 or SCC7 cells were seeded at a density of 1 × 10^5^ cells per well in 6‐well plates and cultured for 24 h. Cells were treated with different concentrations of KNN NPs for 4 h, followed by US combined with U‐PEH for 30 s. DCFH‐DA staining was performed for 30 min at 37 °C, and cells were washed three times with PBS to remove excess DCFH‐DA before fluorescence photographic recording.

For the lived/dead cell staining assay, incubating cultured CT26 or SCC7 cells at a density of 1 × 10^5^ cells well^−1^ in 6‐well plates with different concentrations of KNN NPs for 4 h and treating the cells with US combined with U‐PEH for 30 s. 24 h after the end of the treatment, cells were stained with Calcein‐AM and propidium iodide (PI) for 20 min and washed thoroughly with PBS 3 times.

### In Vivo Cancer Therapy

5 × 10^5^ CT26 mouse cells dispersed in 100 µL of serum‐free 1640 medium were subcutaneously injected to induce tumors. Experiments were performed when tumor volumes reached ≈60–80 mm^3^. Mice were randomly divided into seven groups (n = 5): (I) control; (II) US; (III) U‐PEH; (IV) KNN (intratumoral injection, 10 mg kg^−1^); (V) KNN (intratumoral injection, 10 mg kg^−1^) +US; (VI) U‐PEH+US; (VII) U‐PEH+KNN (intratumoral injection, 10 mg kg^−1^) +US. During the 12‐day treatment period, mice received KNN NPs injections on days 0, 2, and 4, followed by US stimulation (1.0 MHz, 1.5 W cm^−2^, 1 min of stimulation at 30 s intervals for a total stimulation of 5 min) with or without U‐PEH on days 0, 1, 2, 3, and 4 after intratumoral injection of KNN NPs or PBS, at 24 h intervals. The tumor temperature was measured before and after US stimulation. Tumor volume and mouse body weight were recorded every 2 days throughout the treatment period.

All mice were sacrificed on the twelfth day of the experiment, and the major organs (heart, liver, spleen, and kidney) along with tumors were removed. The post‐sections were preserved in 10% formalin for histopathological analysis, including H&E staining to evaluate cellular status and structure, antigenic Ki‐67 staining to ascertain cell growth rate, terminal deoxynucleotidyl transferase dUTP nick labeling (TUNEL) to identify DNA fragmentation, and intratumor DHE staining to identify in vivo ROS production. Meanwhile, the obtained tumor tissues were subjected to transcriptome analysis. Routine blood and blood biochemical analysis were performed on CT26 tumor‐bearing mice on the 12th day after treatment. All measurements (tumor size, body weight, and histological evaluation) were performed by investigators blinded to group allocation. The sample size (n = 5 per group) was determined based on previous similar studies and the preliminary experiments, which demonstrated that this group size is sufficient to detect statistically significant differences in tumor growth between groups.

In addition, when the subcutaneous CT26 tumors reached a volume of 60–80 mm^3^, KNN NPs were injected intratumorally followed by US stimulation. Four h later, the tumors were collected, fixed in 2.5% glutaraldehyde, and processed for bio‐TEM (JEM‐1400FLASH, JEOL) to observe the distribution of NPs. For the investigation of the metabolism of KNN NPs within the tumors, when the CT26 subcutaneous tumors reached 60–80 mm^3^, a single intratumoral injection of KNN NPs and US stimulation were performed. Tumors were harvested at 0, 6, 12, 14, and 18 days after treatment, and the Nb content in tumors was analyzed using ICP‐OES.

### Bioinformatics Analysis

Tumor tissue is collected from U‐PEH+US group and U‐PEH +KNN+ US group of mice (n = 4) for high‐throughput RNA sequencing 12 days after treatment. High‐throughput RNA sequencing libraries were constructed and high‐throughput RNA sequencing performed following the manufacturer's instructions. DEGs were identified between the two groups (|log2FC| ≥1, FDR <0.05) and gene sets GSEA were achieved using GSEA software. DESeq2 software was used to do a gene differential expression analysis between the two groups, and generate informative volcano maps. Next, perform data analysis using R Studio (version 1.3.959).

### Patient‐Derived Tumor Xenograft (PDX) Models

Human head and neck cancer tissue was obtained from West China Hospital, Sichuan University, in compliance with all applicable ethical rules and with approval from the hospital's ethics committee (Grant No.2021‐1673). Surgically excised head and neck tumor tissue was cut into fragments of ≈2 × 2 × 2 mm^3^ and transplanted into the mid‐dorsal region of nude mice. Mice were killed when the size of the tumor reaches ≈200 mm^3^ (P1). Tumor tissue was obtained and minced into fragments of ≈2 × 2 × 2 mm^3^ and injected into mice to construct P2 tumors as described above, subsequent passaged tumors called P3 and P4 were obtained in the same way, and P5 was used for subsequent experiments. When the size of P5 tumor reached ≈100 mm^3^, tumor‐bearing mice were randomly divided into four groups (n = 5): control group, U‐PEH alone group, U‐PEH+US group, and U‐PEH+KNN+US group. Mice in the U‐PEH+KNN+US group were injected with KNN NPs on the 0th, 2nd and 4th days, and were stimulated with U‐PEH combined with US (1.0 MHz, 1.5 W cm^−2^, 5 min) continued for 5 days, and KNN NPs were not injected into the U‐PEH+US group. For the duration of the treatment period, tumor volume and mouse body weight were measured every two days. H&E and fluorescence labeling were performed on mouse tumors and primary organs.

### Statistical Analysis

All data were presented as mean ± SD, with a significant difference of *p* <0.05. *p* values >0.05 was not significant (n.s.). All statistical analyses were performed using Prism Graph‐Pad 9.5 software. The two‐sided Student's t‐test was used for comparison between the two groups. The difference in animal survival rate was calculated using the Kaplan‐Meier technique, and the *P* value was determined using the log‐rank test. The statistical difference was defined as *p <0.05, **p <0.01, ***p <0.001.

## Conflict of Interest

The authors declare no conflict of interest.

## Author Contributions

J.J., H.X., K.C., and Y.S. contributed equally to this work. X.C.P., L.M.J., and J.G.W. conceived and designed experiments; H.Y.X. and X.H. prepared the piezoelectric materials and devices and conducted tests, data collection, and analysis; J.J., K.L.C., Y.L.S., and X.H. conducted in vitro and in vivo experiments; H.Y.X. and J.J. wrote the manuscript; X.C.P., L.M.J., J.G.W., and X.L.H. supervised the work. All authors discussed and commented on the manuscript.

## Supporting information



Supporting Information

## Data Availability

The data that support the findings of this study are available in the supplementary material of this article.
