# Supplementary material for: Synergistic Anti‐Cancer Therapy Through Ultrasound‐Induced Piezocatalytic Therapy and Tumor Treatment Fields
Source: Adv Sci (Weinh). 2025 Oct 14;13(2):e11604. doi: 10.1002/advs.202511604 (PMC12786362; doi:10.1002/advs.202511604)
Supplement: Supplementary file 1 — Supporting Information [file ADVS-13-e11604-s001.docx]

**Synergistic Anti-cancer Therapy through Ultrasound-induced Piezocatalytic Therapy and Tumor Treatment Fields**

Jing Jin^1,3^†, Haoyue Xue^2^†,Keliang Chen^1^†, Yonglin Su^4^†, Xin Hu^1^, Xiaolin Hu^5^, Xing Huang,^2^Laiming Jiang^2^*, Jiagang Wu^2^*, Xingchen Peng^1^*

^1^Department of Biotherapy, Cancer Center, West China Hospital, Sichuan University, Chengdu 610041, China.

^2^College of Materials Science and Engineering, Sichuan University, Chengdu, 610064, China.

^3^Department of Obstetrics and Gynecology, West China Second University Hospital of Sichuan University, Chengdu, Sichuan, China.

^4^Department of Rehabilitation, Cancer Center, West China Hospital, Sichuan University, Chengdu, 610041, China.

^5^West China School of Nursing, West China Hospital, Sichuan University, Chengdu, 610041, China.

*Correspondence: laimingjiang@scu.edu.cn (L. Jiang), msewujg@scu.edu.cn (J. Wu), pxx2014@163.com (X. Peng)

† J. Jin, H. Xue, K. Chen and Y. Su contributed equally to this work.

The PDF includes:

Figure S1-S22


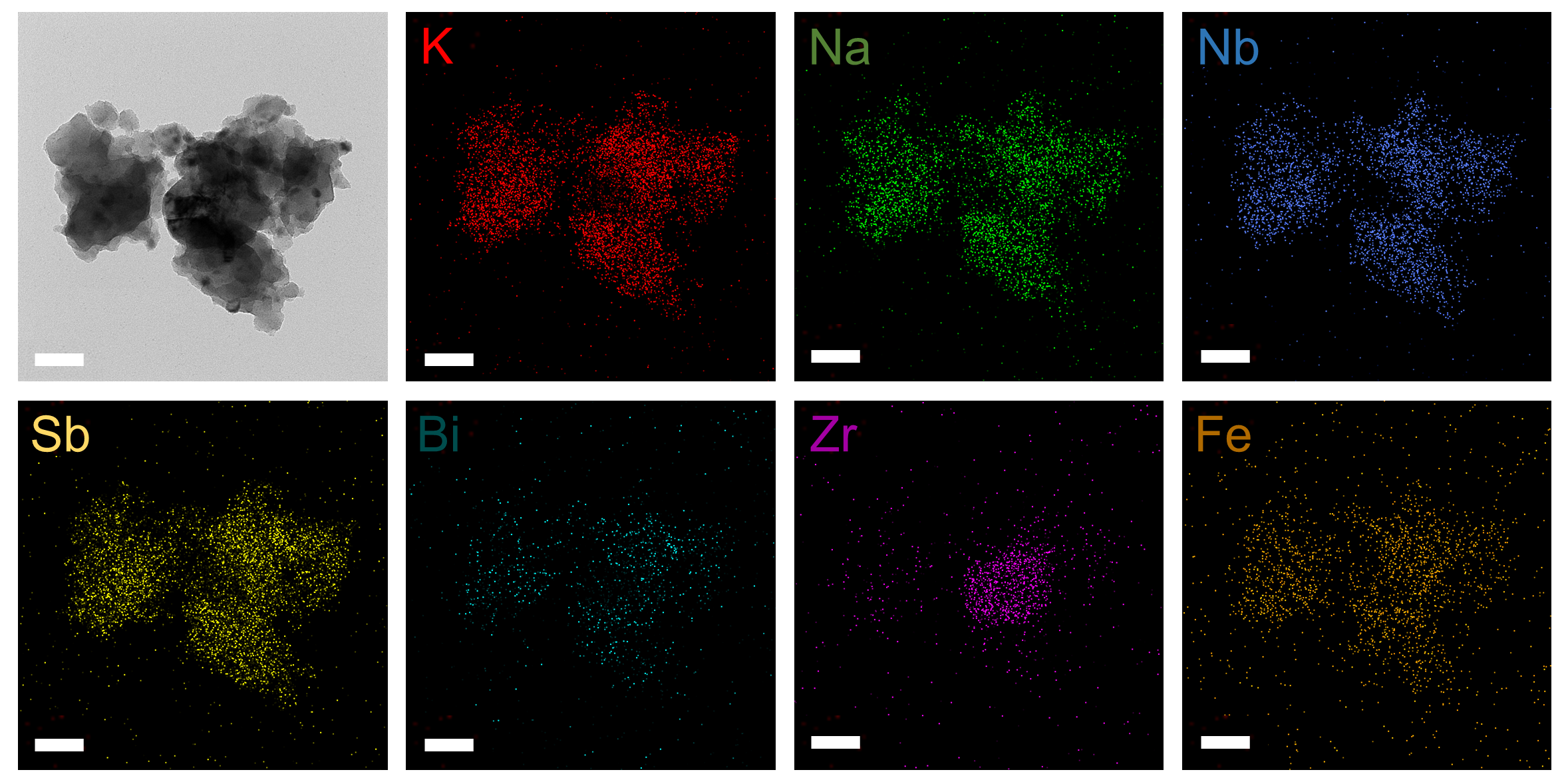


**Figure S1 TEM image and corresponding element mapping images of KNN NPs.** Scale bars, 100 nm.


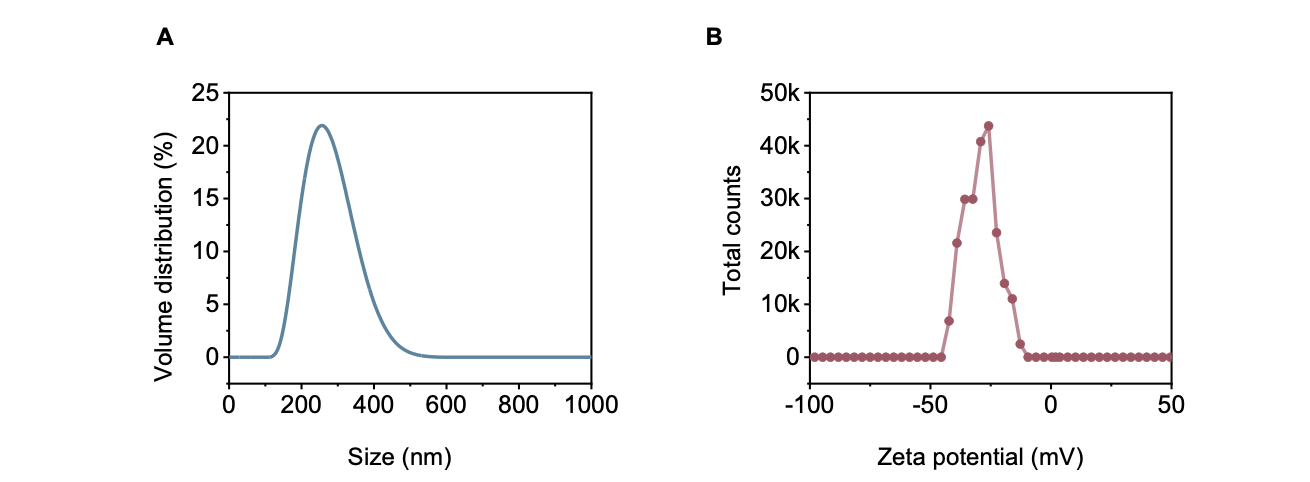


**Figure S2 Dynamic light scattering analysis of KNN NPs.** (A) Particle size distribution. (B) Zeta potential.


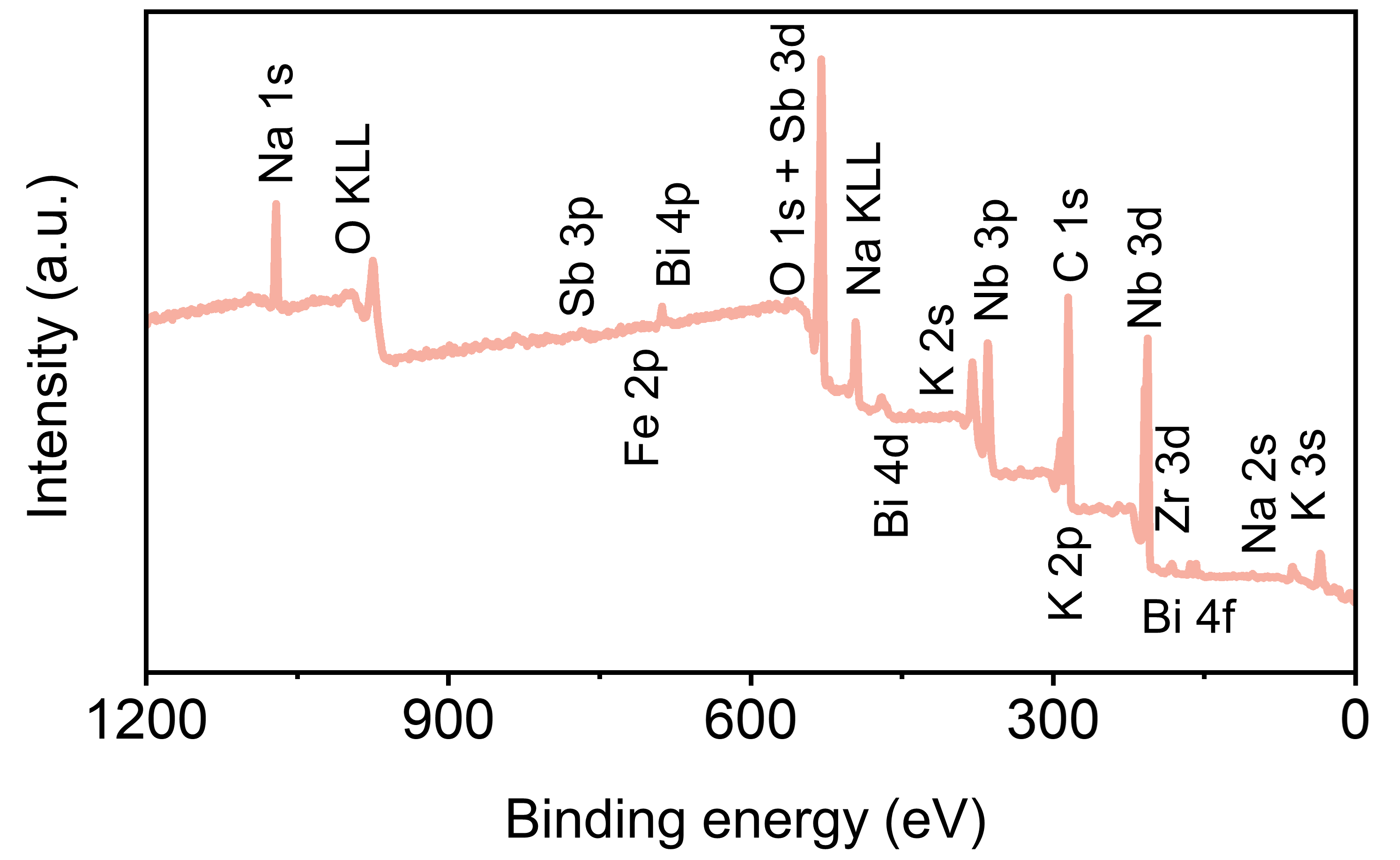


**Figure S3 Wide scan XPS spectra of KNN NPs.**


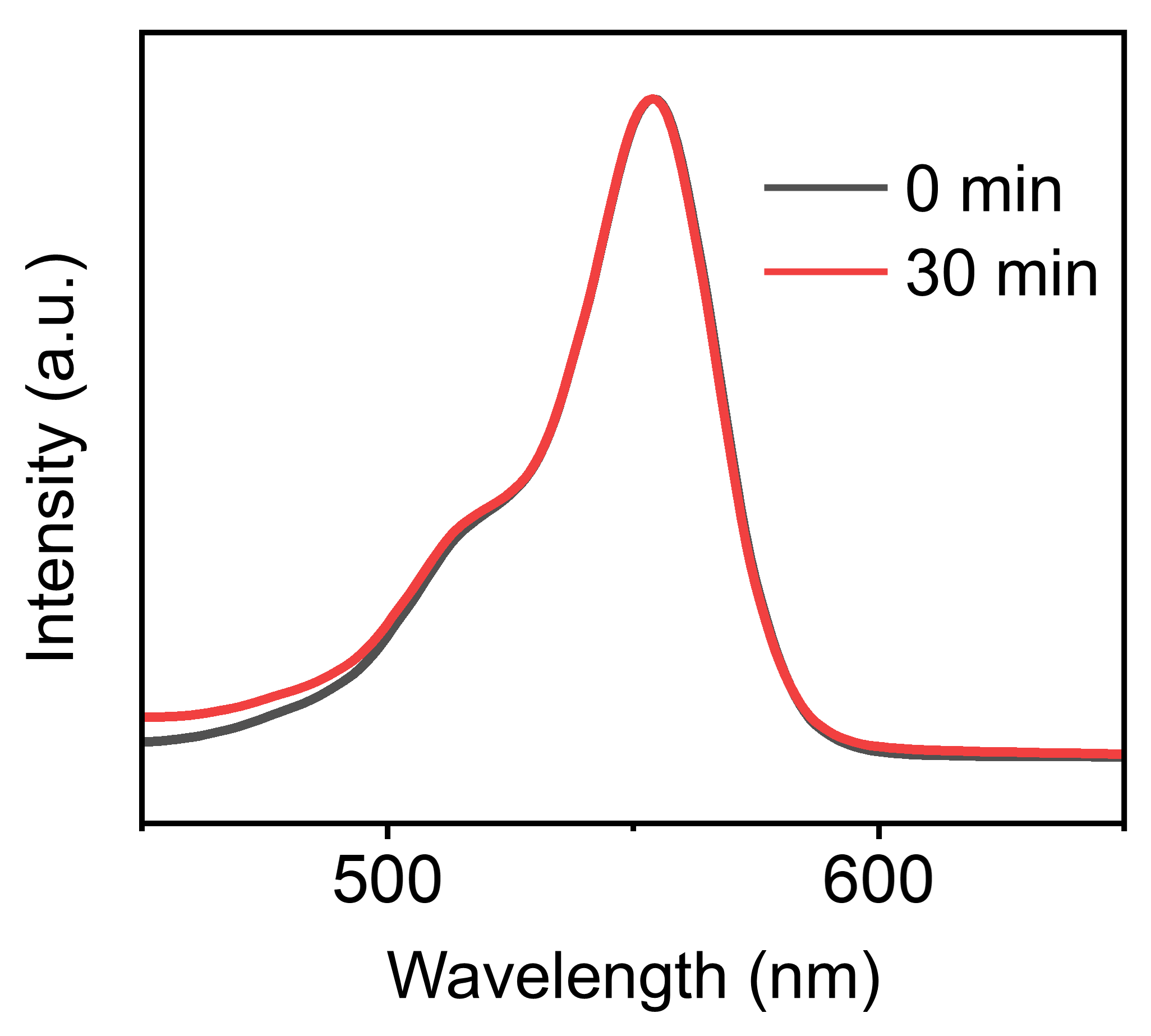


**Figure S4** **Degradation of Rho B under US stimulation for 0 min and 30 min.** US alone caused no significant degradation of Rho B.


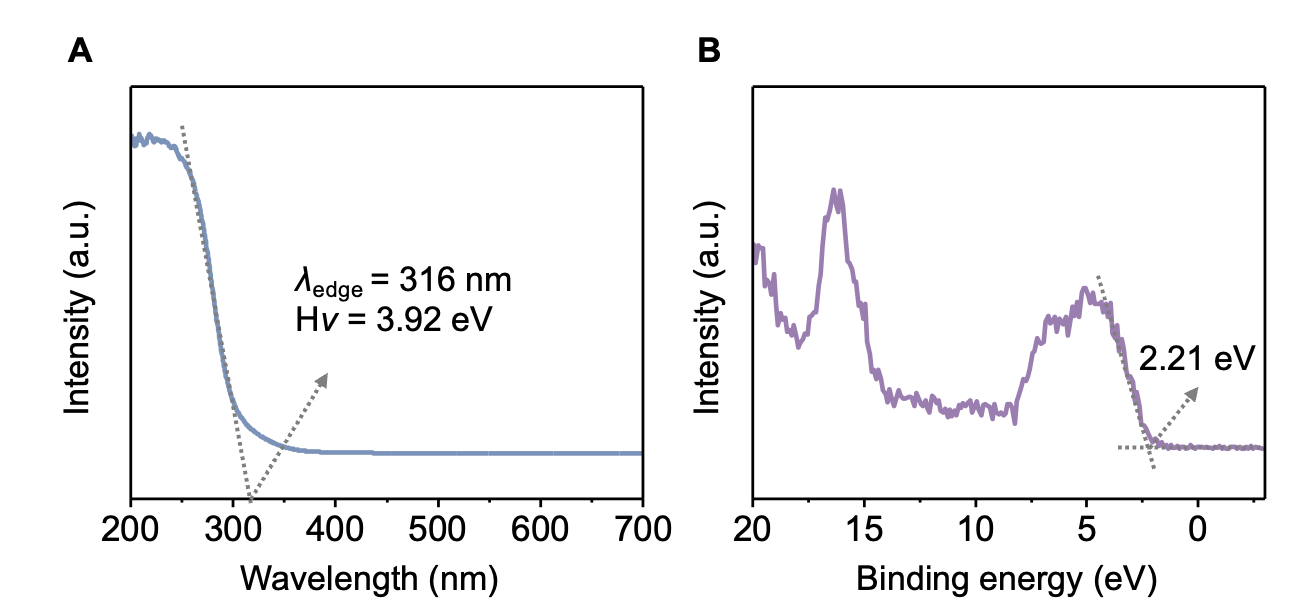


**Figure S5 Characterization of energy band structure of KNN NPs.** (A) UV-vis absorption spectra. (B) XPS valence band spectra.


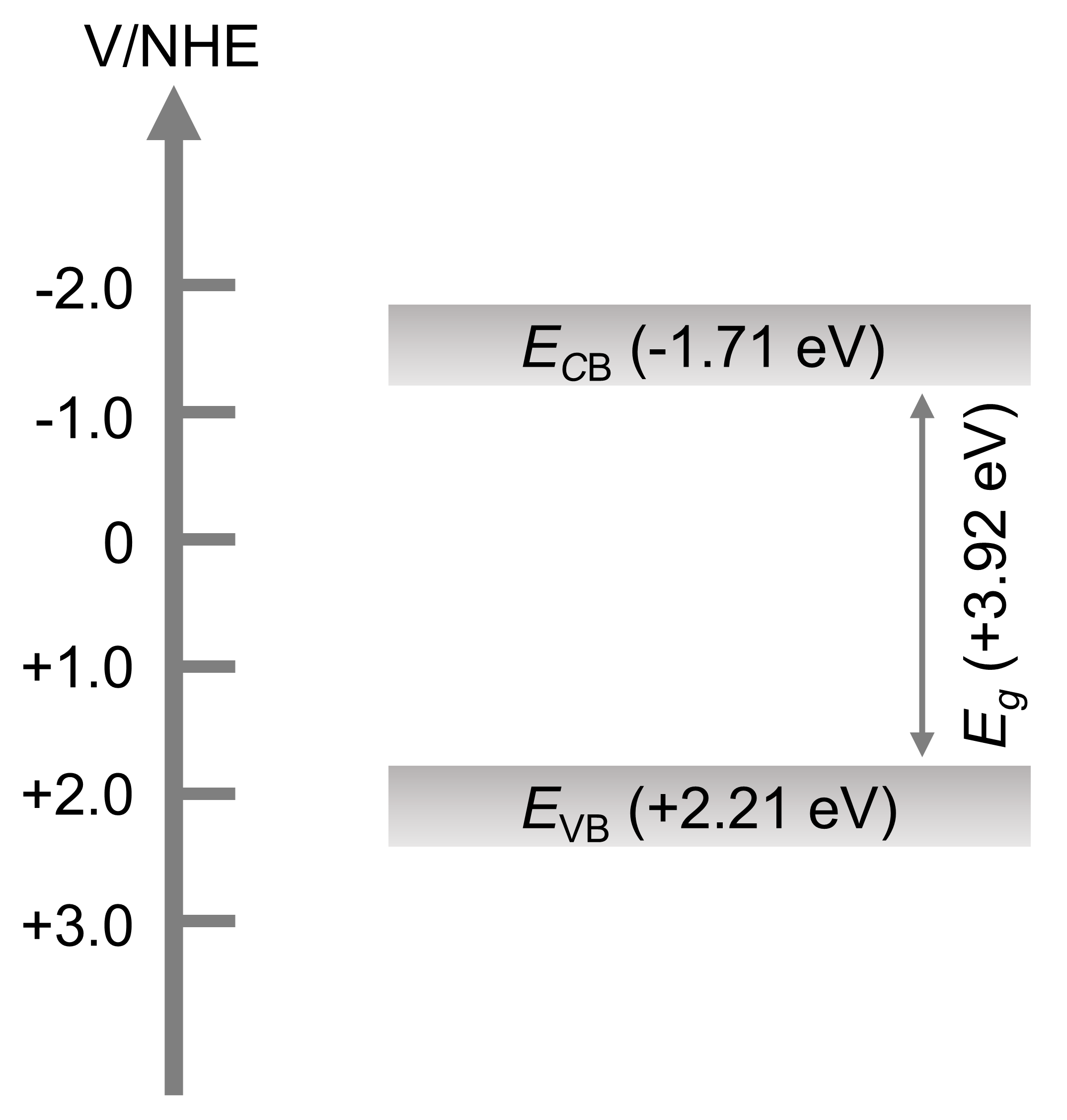


**Figure S6 Energy band structure of KNN NPs.**


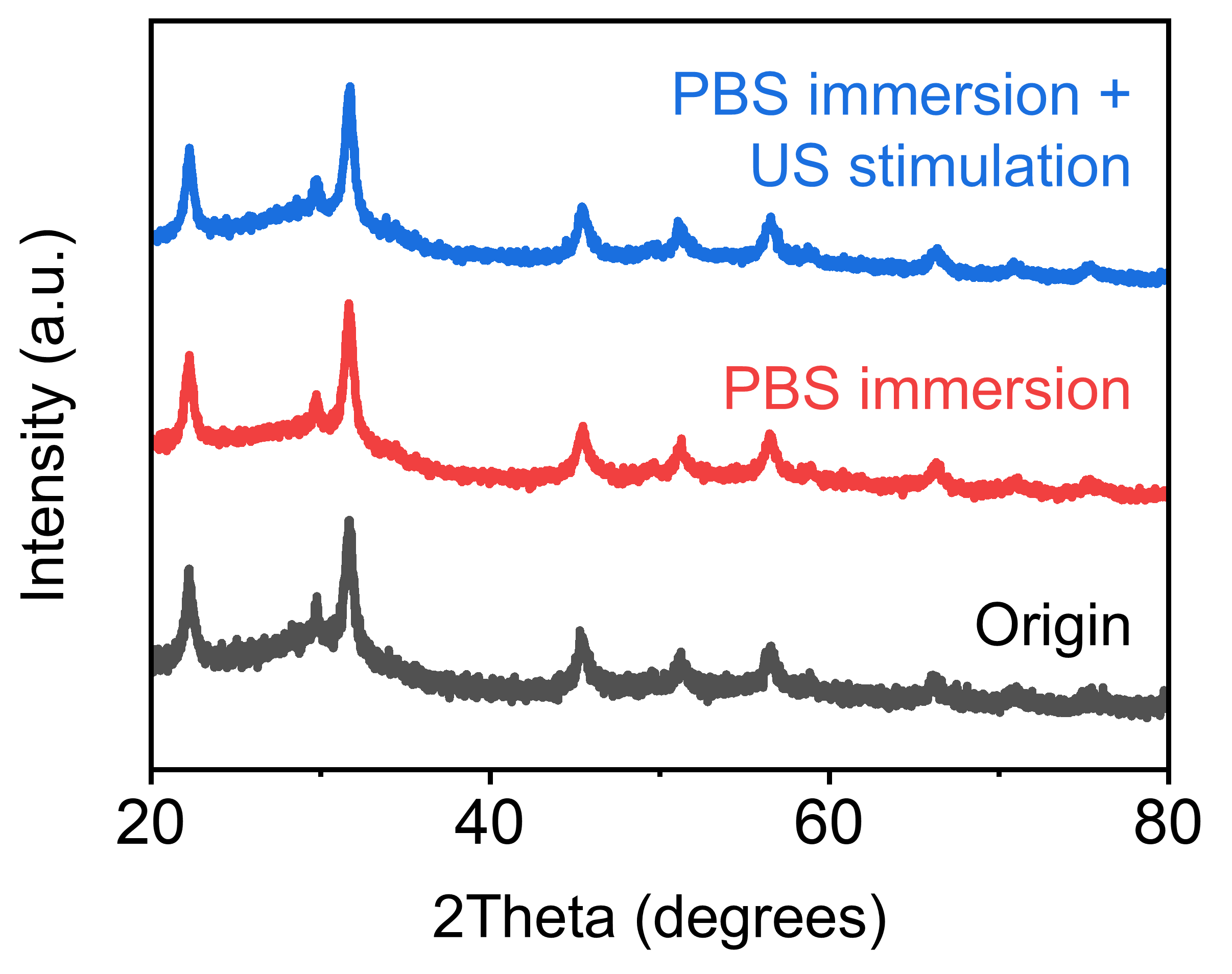


**Figure S7 XRD results of KNN NPs post different treatments.** The structure of KNN NPs remained unchanged after 12 days of immersion in PBS at 37°C, with or without daily US stimulation (5 min).


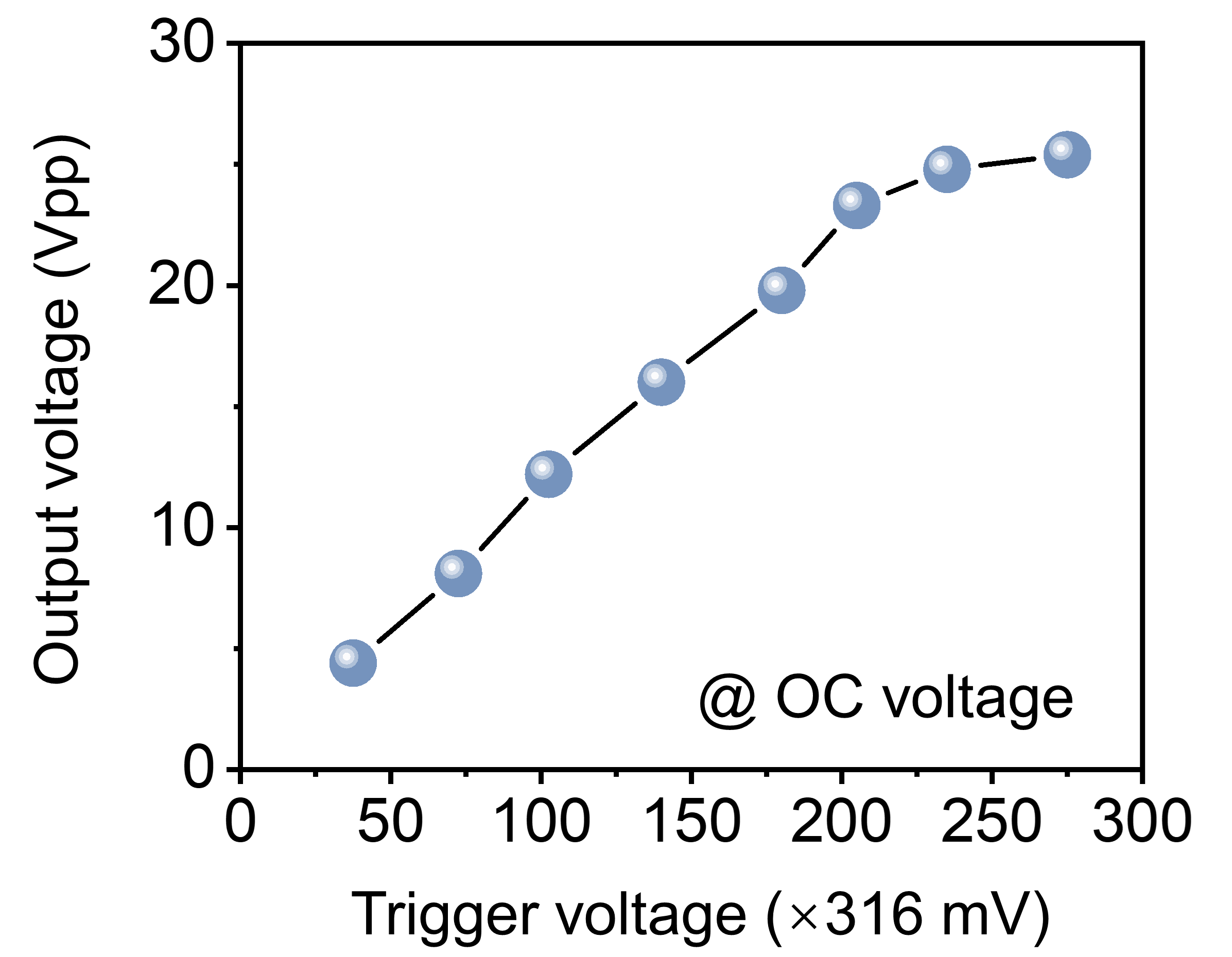


**Figure S8 Output voltages of the U-PEH against trigger voltages.** The output voltages are positively correlated with the trigger voltages and increase as the trigger voltage increases.


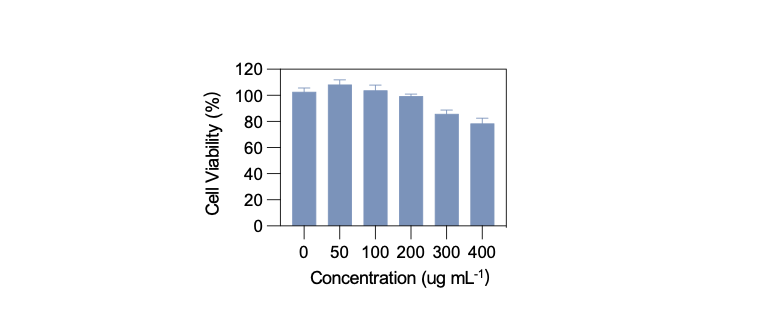


**Figure S9** Cell viability of Hacat cells after 24 h treatment with different concentrations of KNN NPs (n = 3).


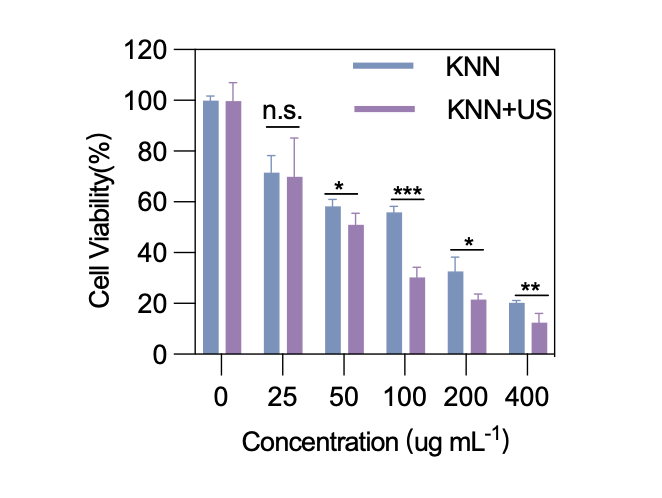


**Figure S10** Cytotoxicity of KNN NPs on SCC7 cells with ultrasound irradiation by CCK8 assay (n = 4). The two-sided Student's t-test was utilized to determine statistical significance. (**P* < 0.05, ***P* < 0.01, ****P* < 0.001).

**
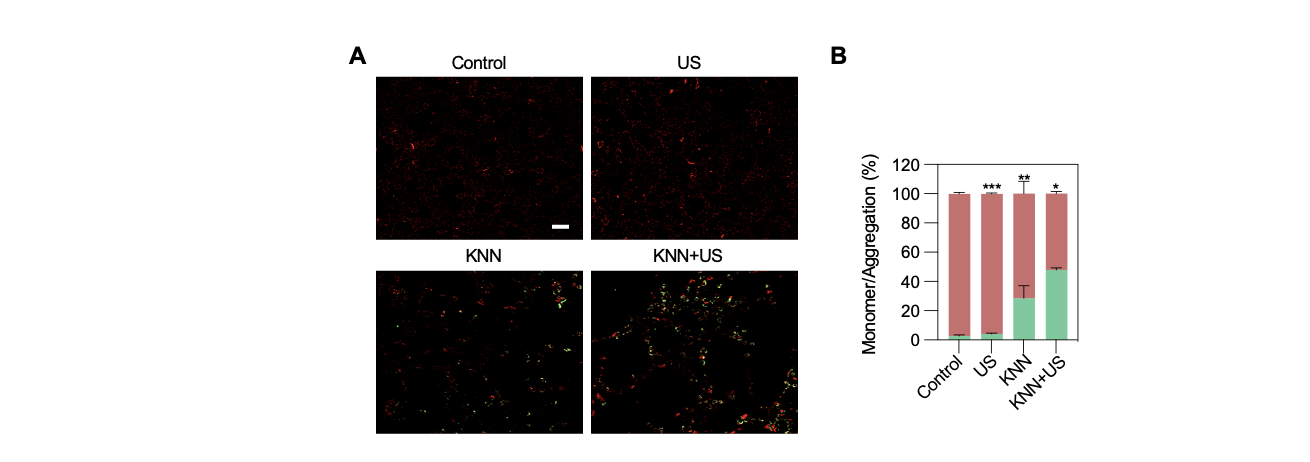
**

**Figure S11** (A) Fluorescence images SCC7 cells stained with JC- 1 after various treatments. scale bar, 50 μm. (B) Green/red fluorescence ratios of JC-1 stained SCC7 cells following various treatments (n = 3). The two-sided Student's t-test was utilized to determine statistical significance. (**P* < 0.05, ***P* < 0.01, ****P* < 0.001).


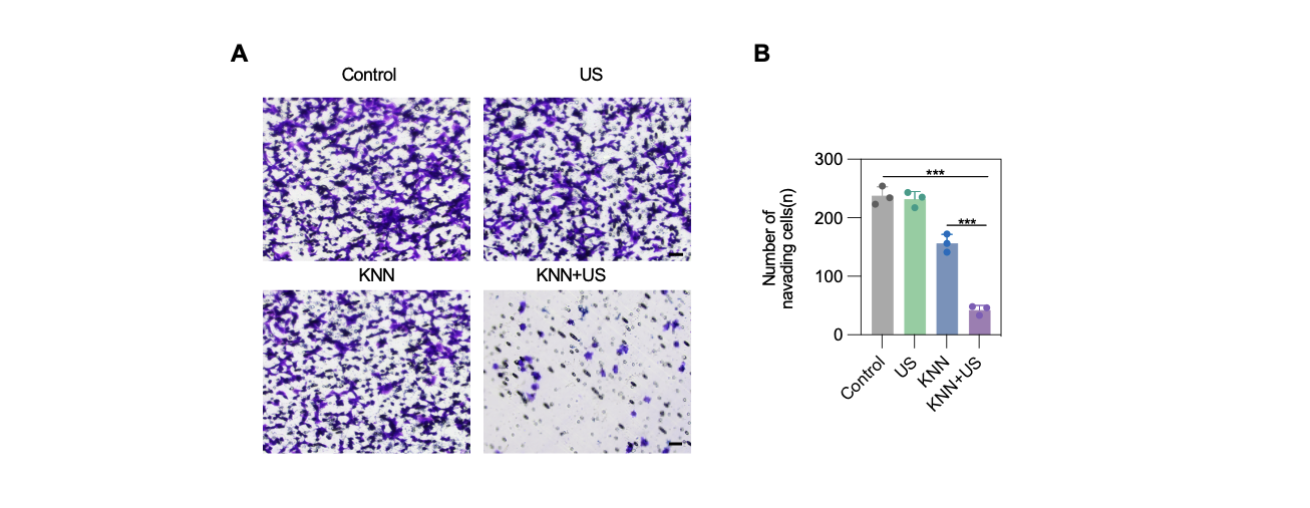


**Figure S12** (A) Transwell assay of invasion ability of SCC7 cells after different treatments. Scale bars, 50 μm. (B) Quantitative analysis of number of navading SCC7 cells after different treatments (n = 3). The two-sided Student's t-test was utilized to determine statistical significance. ****P* < 0.001.

**
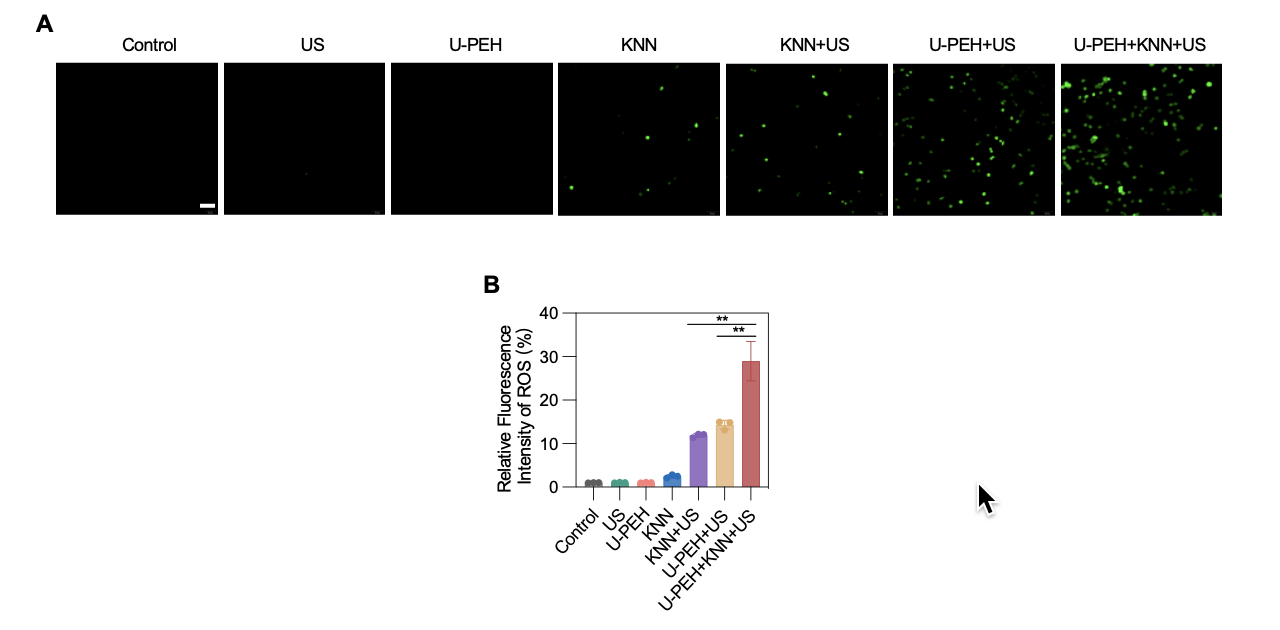
**

**Figure S13** (A,B) Fluorescence images (A) and quantitative analysis (B) of DCFH-DA in SCC7 cells after different treatments (n = 3). Scale bar, 50 μm. The two-sided Student's t-test was utilized to determine statistical significance. ***P* < 0.01.


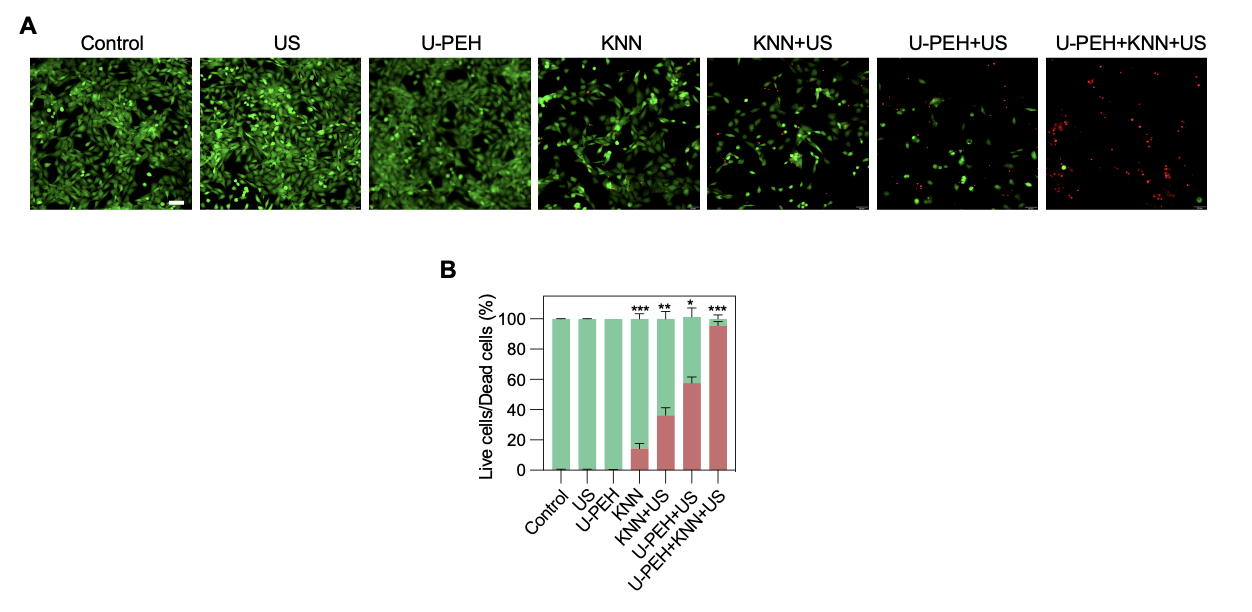


**Figure S14** (A) Live (green) and dead (red) assay of SCC7 cells treated with KNN NPs combined with/without U-PEH under ultrasound irradiation. Scale bar, 50 μm. (B) Quantitative analysis of red/green fluorescence ratios in SCC7 cells (n = 3). The two-sided Student's t-test was utilized to determine statistical significance. (**P* < 0.05, ***P* < 0.01, ****P* < 0.001).


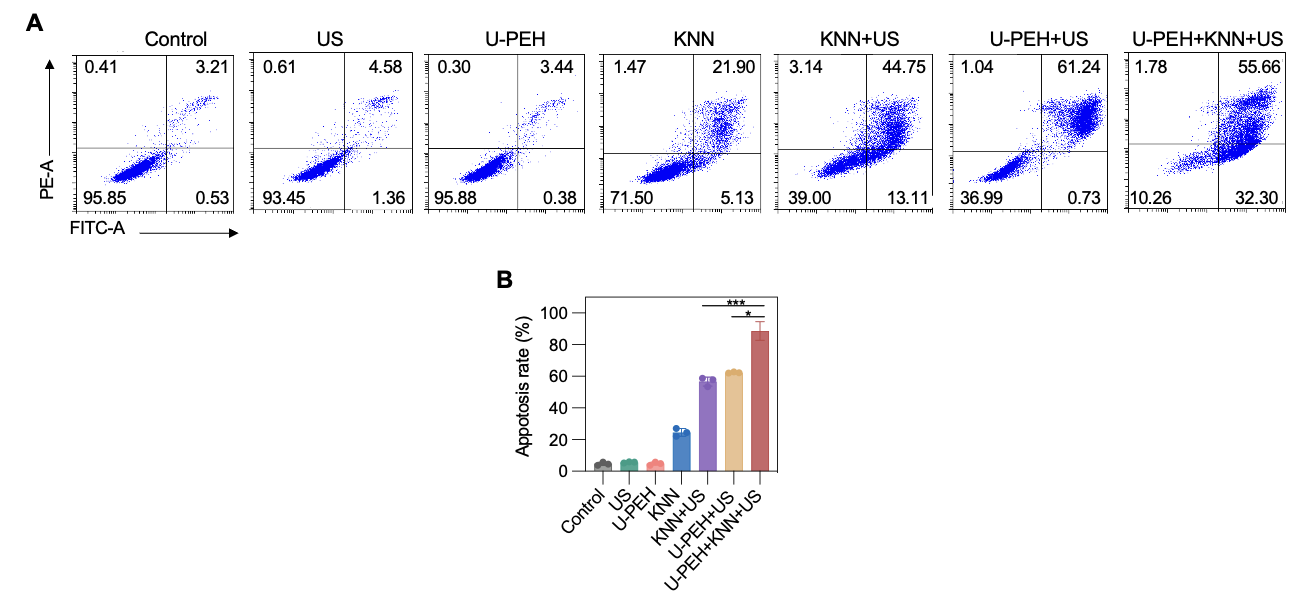


**Figure S15** (A) Apoptosis of SCC7 cells after 48 hours of treatment with Control, US, U-PEH, KNN, KNN+US, U-PEH+US, and U-PEH+KNN+US. (B) Corresponding quantification statistics of apoptosis in SCC7 cells after 48h different treatments (n = 3). The two-sided Student's t-test was utilized to determine statistical significance. (**P* < 0.05, ***P* < 0.01, ****P* < 0.001).


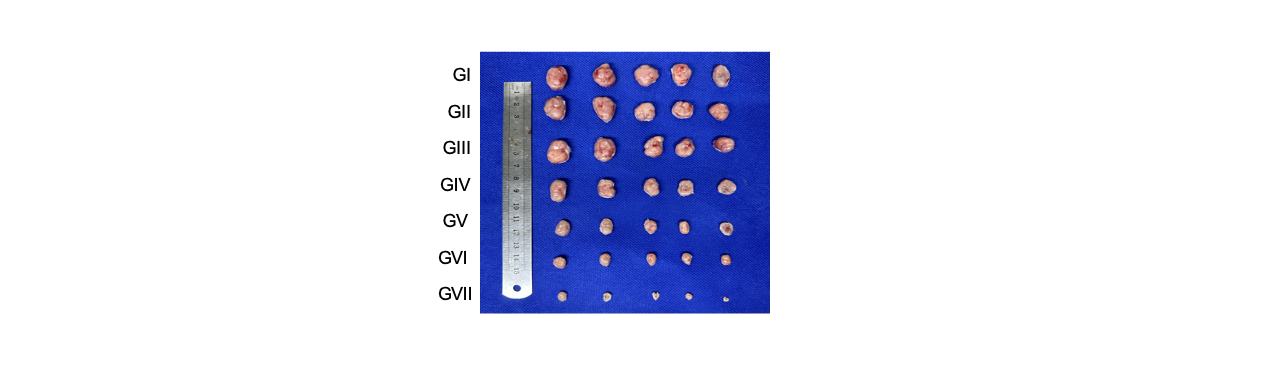


**Figure S16** Digital photos of excised tumors after various treatments (n = 5).


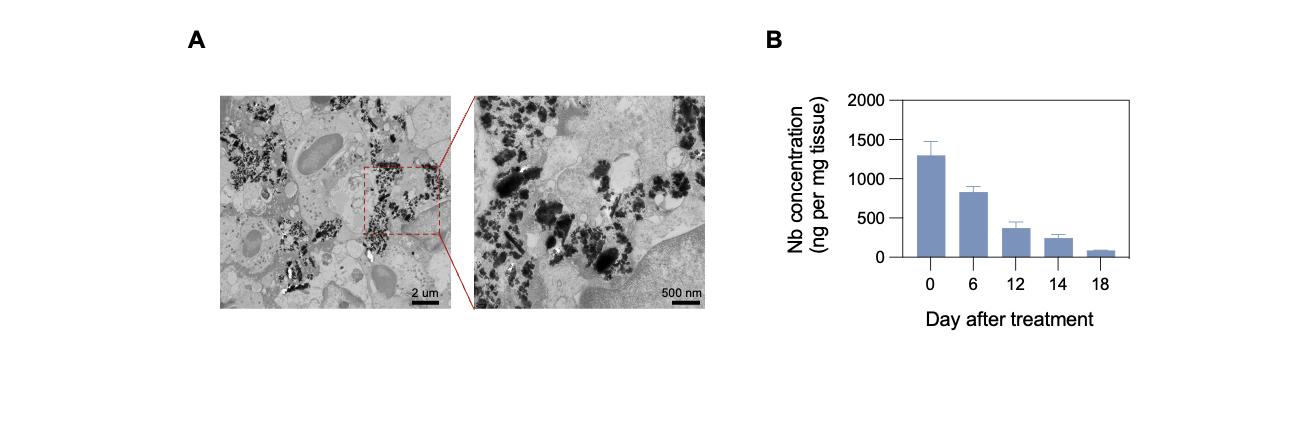


**Figure S17** (A) Representative biological TEM images illustrating the distribution of KNN NPs within tumors following US-excited KNN combined with U-PEH treatment. (B) Quantification of Nb concentration in tumors over time following a single KNN treatment was measured by ICP-OES, demonstrating the retention of KNN NPs at the tumor site throughout the treatment period (n = 3). Data are presented as mean ± SD.


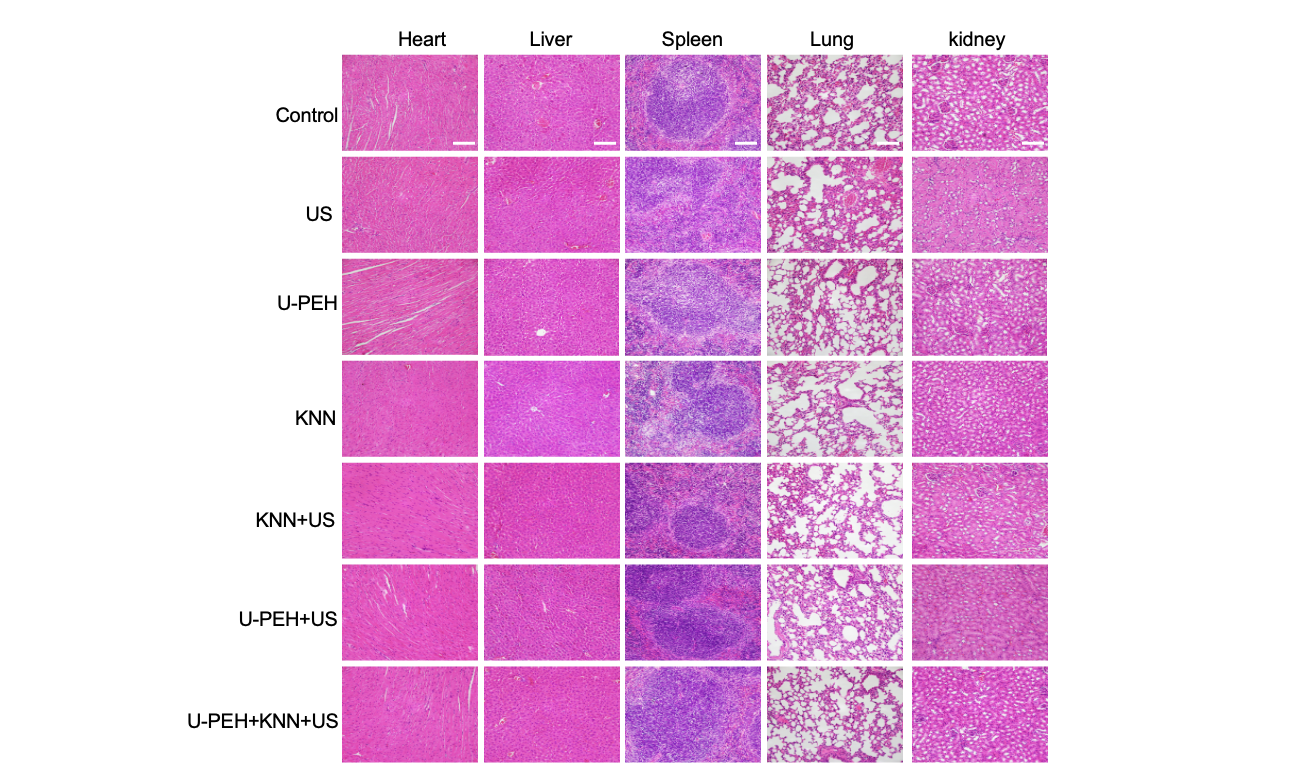


**Figure S18** H&E staining of major organs (heart, liver, spleen, lung and kidney) from mice treated with control, US, U-PEH, KNN, KNN+US, U-PEH+US, and U-PEH+KNN+US at the end point. Scale bars, 50 μm.


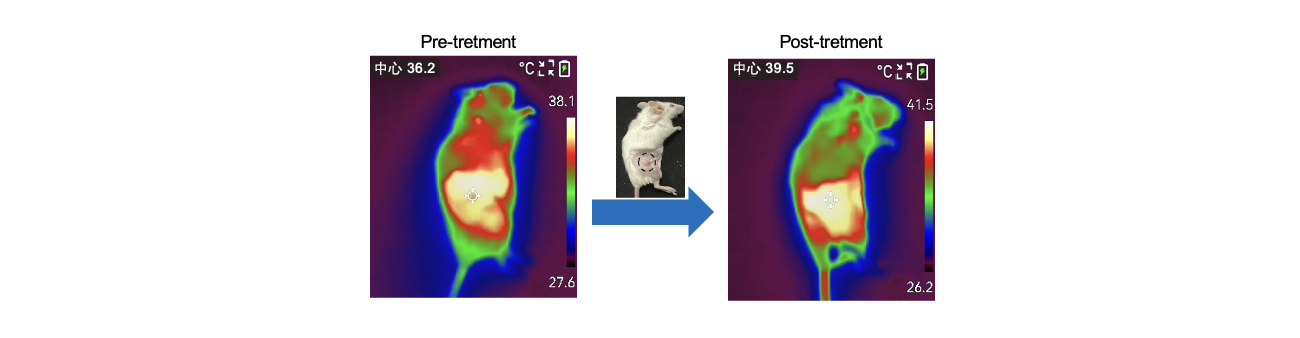


**Figure S19** Representative infrared thermal images of tumor-bearing mice before and after U-PEH+US treatment.


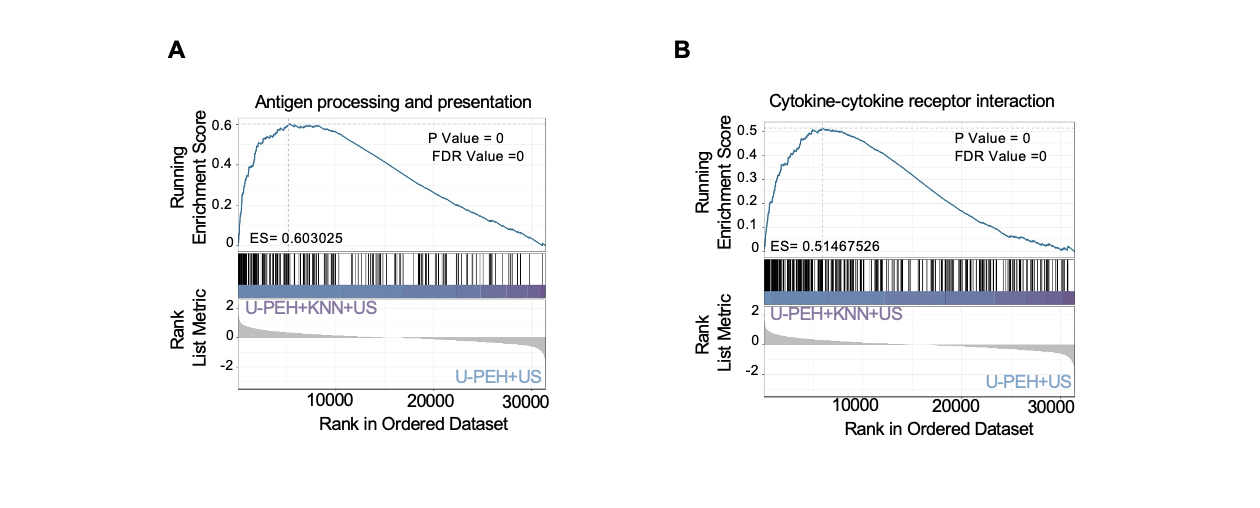


**Figure S20** GSEA enrichment analysis of the DEGs following U-PEH+KNN+US therapy in comparison to U-PEH+US treatment.


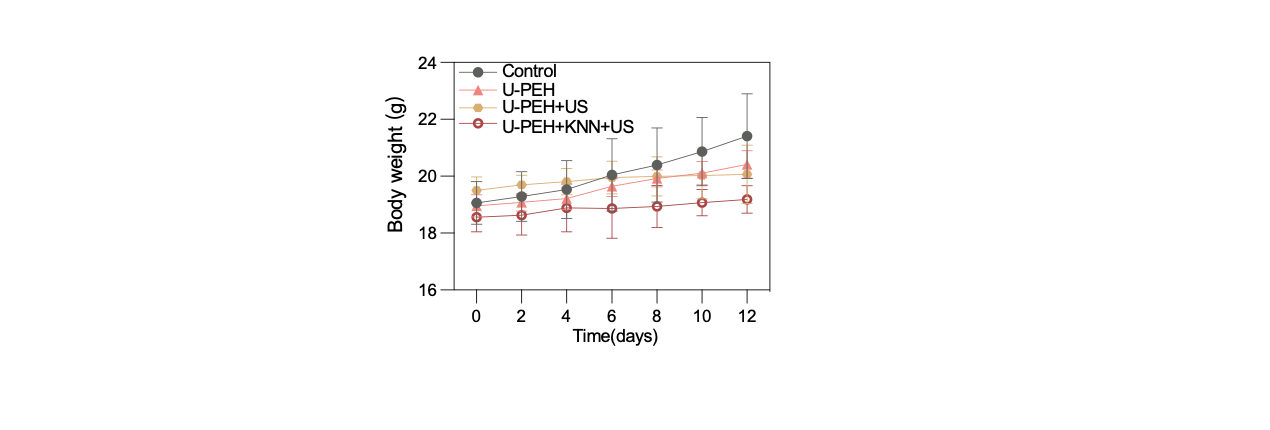


**Figure S21** The body weight of mice undergoing therapy (n = 5).


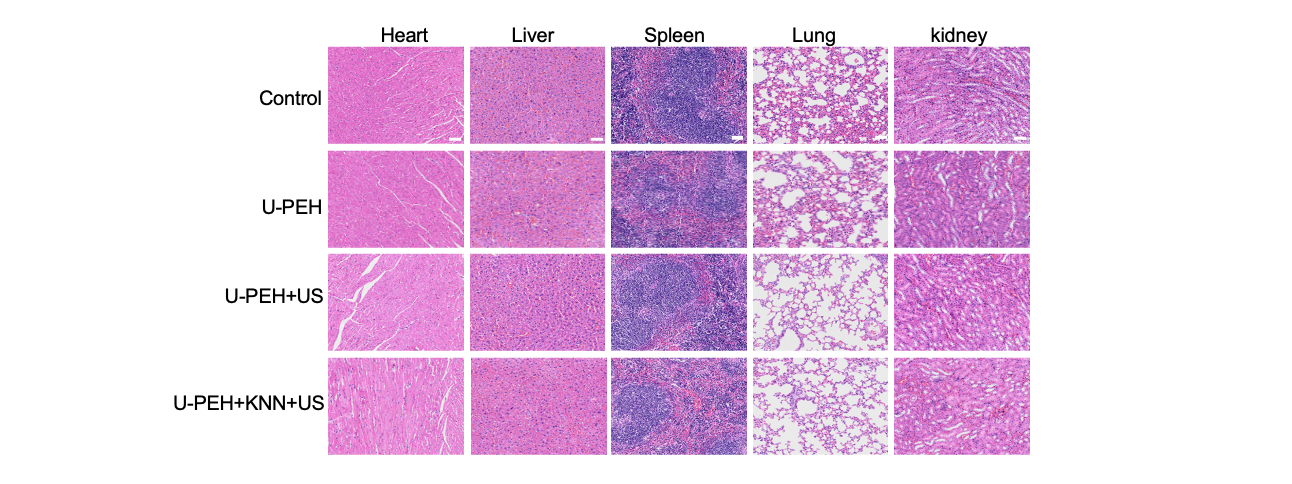


**Figure S22** H&E staining of major organs (heart, liver, spleen, lung and kidney) from HNSCC PDX-bearing mice treated with Control, U-PEH, U-PEH+US, and U-PEH+KNN+US at the end point. Scale bars, 100 μm.
